# Cumulative Burden of Digital Health Technologies for Patients With Multimorbidity: A Systematic Review

**DOI:** 10.1001/jamanetworkopen.2025.7288

**Published:** 2025-04-25

**Authors:** Ngan Thi Thuy Phi, Victor M. Montori, Marleen Kunneman, Philippe Ravaud, Viet-Thi Tran

**Affiliations:** 1 Université Paris Cité and Université Sorbonne Paris Nord, INSERM, INRAE, Center for Research in Epidemiology and Statistics (CRESS), Paris, France; 2 Knowledge and Evaluation Research Unit, Mayo Clinic, Rochester, Minnesota; 3 Department of Biomedical Data Sciences, Section of Medical Decision Making, Leiden University Medical Center, Leiden, the Netherlands; 4 Centre d’epidémiologie clinique, AP-HP, Hôpital Hôtel Dieu, Paris, France; 5 Department of Epidemiology, Mailman School of Public Health, Columbia University, New York, New York

## Abstract

**Importance:**

Digital health technologies (DHTs) aiming to monitor, treat, and manage diseases can be prescribed for patients with multimorbidity; yet most DHTs are designed for individual conditions or problems, while approximately half of patients with chronic conditions have multiple chronic conditions.

**Objectives:**

To identify DHTs approved by the US Food and Drug Administration (FDA) or listed in the Organisation for the Review of Care and Health Apps (ORCHA) library and prescribable for a hypothetical patient with 5 conditions and to model the number of DHTs this patient should be prescribed to receive benefits health professionals considered important.

**Evidence Review:**

The FDA databases (Premarket Notification 510(k), Premarket Approval, and De Novo) and the ORCHA App Library from National Health Service Somerset were systematically searched for DHTs registered or updated between January 1, 2019, and December 31, 2022, that could be prescribed to a hypothetical woman with 5 chronic conditions (type 2 diabetes, hypertension, chronic obstructive pulmonary disease, osteoporosis, and osteoarthritis). After abstracting each DHT’s elementary functions (ie, simple and delineated features to monitor, treat, and/or manage conditions), an assessment was undertaken to determine the fewest DHTs this hypothetical patient should be prescribed to receive benefit from digital functions health professionals considered important.

**Findings:**

A total of 148 DHTs were identified (68 [46%] from FDA databases), of which 96 (65%) involved devices and 52 (35%) were standalone health apps. Only 5 DHTs (3.4%) were intended for 2 or more conditions. DHTs offered 140 elementary functions, ranging from recording, tracking, or visualizing health parameters to providing information to digital therapeutics with just-in-time interventions. The hypothetical patient would need to be prescribed up to 13 apps and 7 devices (a blood pressure monitor, a smartwatch, a pulse oximeter, a connected weight scale, a sensor-attached inhaler to monitor adherence, a lung function monitor, and a blood glucose sensor) to receive benefits from 28 functions at least 3 of 5 health professionals considered important.

**Conclusions and Relevance:**

This systematic review found that almost all prescribable DHTs were developed for a single condition or problem. Thus, patients with multiple chronic conditions would have to routinize many DHTs concurrently in daily life to benefit from digital functions health professionals considered important.

## Key Points

**Question:**

What digital health technologies (DHTs) are available for patients with multimorbidity and how many individual DHTs would a hypothetical patient need to benefit?

**Findings:**

This systematic review assessed 148 DHTs approved by the US Food and Drug Administration or vetted by the Organisation for the Review of Care and Health Apps and prescribable for a hypothetical patient with 5 chronic conditions (type 2 diabetes, hypertension, chronic obstructive pulmonary disease, osteoporosis, and osteoarthritis). Less than 5% of DHTs were developed for 2 or more conditions or problems, and this patient would need to use at least 15 DHTs to benefit from digital functions clinicians considered important.

**Meaning:**

These findings suggest that patients with multimorbidity would have to routinize many DHTs concurrently in daily life to benefit from digital functions clinicians considered important.

## Teaser

This systematic review identifies digital health technologies (DHTs) prescribable for a hypothetical patient with 5 conditions and models the number of DHTs this patient would need to use to receive benefits health professionals considered important.

## Introduction

Digital health technologies (DHTs) use computing platforms, connectivity, software, and sensors for health care.^1^ They offer the possibility to monitor patients remotely, continuously, and passively using wearable devices, to detect important events from these data using artificial intelligence (AI) and/or machine learning, and to deliver timely (also known as just-in-time) personalized interventions at home and at low cost.^2,3,4,5,6^ DHTs support transitioning care, happening solely during short and episodic encounters with clinicians at the point of care, to care at the point of life, where physiological and behavioral patterns are monitored and used to make clinical decisions.^7^

Yet the adoption of DHTs in the treatment of patients with chronic conditions is limited.^8^ Several factors may influence the adoption of DHTs.^9,10,11^ We hypothesized one potential reason might be that most DHTs are designed for individual conditions, while approximately 50% of patients with chronic conditions have multiple chronic conditions (eg, 82% of patients with diabetes have ≥1 additional chronic conditions).^3,12,13^ Such a disease-by-disease approach might result in patients having to use multiple apps and devices for their different needs, which may be unacceptable, too burdensome, and ultimately ineffective.^14^

We identified all DHTs prescribable for a hypothetical patient with 5 chronic conditions (type 2 diabetes, hypertension, chronic obstructive pulmonary disease [COPD], osteoporosis, and osteoarthritis), and assessed the number of DHTs this patient should be prescribed to receive benefits from functions health professionals considered important. We chose to evaluate devices involving hardware and standalone apps together to reflect patients’ perspective, as patients would have to cope with tasks from all DHTs, regardless their nature.

## Methods

For this systematic review without meta-analysis, we used the Preferred Reporting Items for Systematic Reviews and Meta-analyses (PRISMA) reporting guideline when applicable.^15^ This study followed 3 steps. First, we identified all DHTs prescribable to a hypothetical woman aged 79 years, with 5 common comorbid diseases: type 2 diabetes, hypertension, COPD, osteoporosis, and osteoarthritis, all of moderate severity.^16^ The hypothetical patient was inspired by the patient from the study by Boyd et al^16^ and has diseases prevalent in older individuals that are usually managed in primary care. Second, we abstracted the elementary functions of the identified DHTs (ie, what DHTs can do to help monitor, treat, and/or manage diseases). Finally, we assessed the number of DHTs the hypothetical patient would need to be prescribed to receive benefits from all functions and important functions, according to health professionals.

### Identifying DHTs

We systematically searched 3 FDA databases, Premarket Notification 510(k), Premarket Approval, and De Novo, and the Organisation for the Review of Care and Health Apps (ORCHA) App Library from National Health Service (NHS) Somerset, for approved or recommended DHTs registered or updated between January 1, 2019, and December 31, 2022.^17,18,19,20^ The ORCHA App Library from NHS Somerset was used instead of the American College of Physicians version because the latter was examined in a preliminary stage and included fewer than 30 apps at the time of the study.^21^ Our search strategy is detailed in eMethods 1 in Supplement 1.

We defined DHTs as all software as a medical device (SaMD)^22^; implanted, wearable, external, or environmental medical devices driven by software; and health applications (run on smartphones or computers). To be eligible, the DHTs had to be applicable for the hypothetical patient (ie, they were intended for monitoring, treating, and/or managing diabetes, hypertension, COPD, osteoarthritis, and/or osteoporosis and intended for older female patients), intended to be used outside of clinical settings, and intended to be used by patients. Exclusion criteria are detailed in eMethods 2 in Supplement 1. The selection involved 2 reviewers (N.T.T.P. and V.-T.T.) and is detailed in eMethods 3 in Supplement 1. For each DHT included in the study, 1 reviewer (N.T.T.P.) extracted its name, manufacturer or developer, last updated time (or released if the first time), target disease (as categorized by the FDA or ORCHA), type of use (over the counter or prescription), and cost (entirely free, in-app purchases, or paid).

### Abstracting Elementary Functions Provided by DHTs

For each DHT included, 1 reviewer (N.T.T.P.) abstracted all its elementary functions based on the information provided in FDA product summaries and developer description of DHTs retrieved from the ORCHA app library from NHS Somerset. An elementary function was defined as a simple and delineated feature to monitor, treat, and/or manage conditions, such as providing information on diabetes or monitoring heart rate. The level of granularity of elementary functions was discussed in several meetings with health professionals and researchers from Mayo Clinic (US; V.M.), Leiden University Medical Center (the Netherlands; M.K.), and Université Paris Cité (France; N.T.T.P., V.-T.T.).

Elementary functions were then grouped by similarity into 8 categories: (1) recording, tracking, or visualizing health-associated parameters; (2) providing information or education; (3) maintaining motivation; (4) communicating with professionals; (5) communicating with coaches, peers, and loved ones; (6) providing digital therapeutics with just-in-time interventions (ie, digital therapeutic intervention that combines remote monitoring tools and algorithms to personalize the delivery of specific interventions at the right time); (7) providing digital therapeutics without just-in-time interventions; and 8) other functions that did not fall into these categories. Elementary functions were assessed by 5 health professionals (2 internal medicine specialists, 1 pulmonologist, and 2 general practitioners). Each health professional was presented the list of elementary functions abstracted previously and assessed whether each function was useful for most patients similar to the hypothetical patient (hereafter, *important*), useful only in specific cases, or not useful for patients similar to the hypothetical patient. Health professionals did not know which functions were present in which DHTs (eMethods 4 in Supplement 1).

### Statistical Analysis

We generated 2 prescriptions of DHTs for the hypothetical patient. The first prescription, the maximalist prescription, was a combination of the fewest DHTs the hypothetical patient would need to use to benefit from all elementary functions identified. The second prescription, the parsimonious prescription, was a combination of the fewest DHTs the patient would need to use to benefit from elementary functions considered important by at least 3 of 5 health professionals. We also presented as sensitivity analysis the prescription of the fewest DHTs the patient would need to use to benefit from elementary functions considered important by at least 4 of 5 health professionals.

We used a greedy algorithm to solve the combinatorial problem of generating prescriptions of DHTs. In short, the algorithm first prescribed the DHT covering the most functions, then it prescribed a second DHT covering the most functions among those remaining (ie, unprescribed precedingly), and so forth.

Finally, we generated hypothetical patients with all possible combinations of 1, 2, 3, or 4 conditions among those of the hypothetical patient (diabetes, hypertension, COPD, and chronic pain management) and who could (or not) need help to stop smoking, to organize care, and to monitor general health parameters (such as temperature). We then assessed the fewest DHTs these hypothetical patients should use to benefit from functions considered important by at least 3 of 5 health professionals.

Statistical analyses were performed using R software version 4.2.0 (R Project for Statistical Computing). Data were analyzed from January 16 to June 13, 2024.

## Results

### Identifying Relevant DHTs

**Figure 1.**
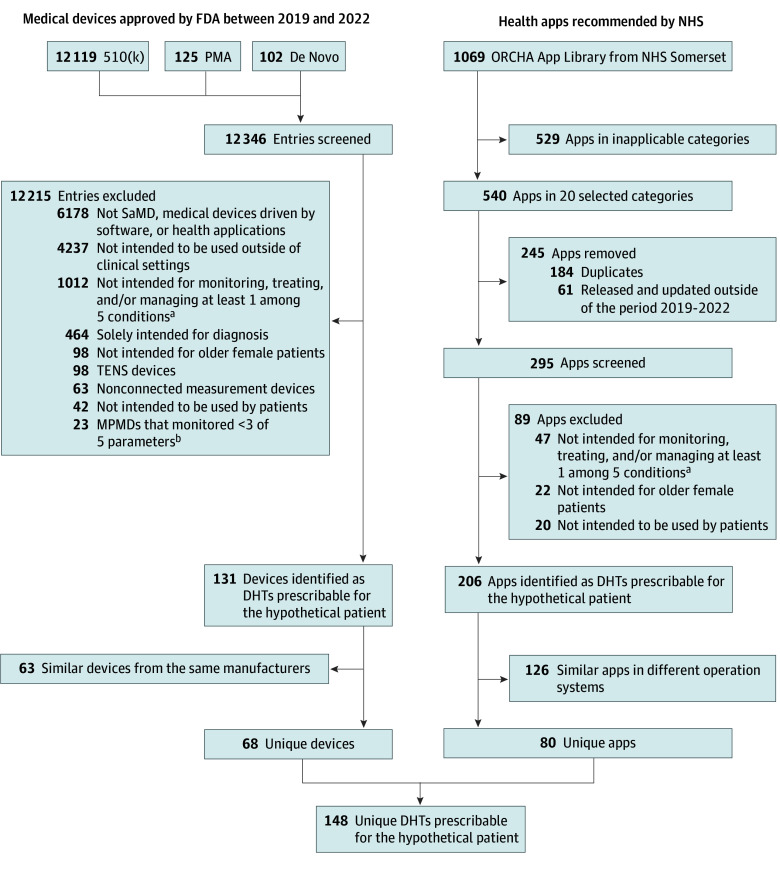
Flowchart for the Selection of Digital Health Technologies (DHTs) Prescribable for a Hypothetical Patient With 5 Chronic Conditions FDA indicates US Food and Drug Administration; MPMDs, multiparameter monitoring devices; NHS, UK National Health Service; ORCHA, Organisation for the Review of Care and Health Apps; PMA, premarket approval; SaMD, software as a medical device; TENS, transcutaneous electrical nerve stimulation. ^a^Includes diabetes, hypertension, chronic obstructive pulmonary disease, osteoarthritis, and osteoporosis. ^b^Includes blood glucose, blood pressure (systolic and diastolic), heart rate (or pulse rate), pulse oximeter, and weight.

We screened 12 346 entries in the FDA databases and 540 entries in the ORCHA App Library and identified 148 unique DHTs (68 [46%] from FDA databases) that could be prescribed to the hypothetical patient (Figure 1). Most DHTs were intended for monitoring, treating, and/or managing diabetes (57 DHTs [39%]), hypertension (25 DHTs [17%]), and COPD (20 DHTs [14%]) (Table 1). In addition to condition-specific DHTs, 58 DHTs (39%) aimed to help users have a healthy lifestyle (eg, smoking cessation, healthy nutrition), and 12 DHTs (8%) aimed to help users with pain management. Of all DHTs, 143 DHTs (97%) were intended for single conditions and problems, and 127 DHTs (86%) were accessible over-the-counter.

**Table 1.** Characteristics of DHTs Prescribable to the Hypothetical Patient With 5 Comorbidities^a^

Characteristic	DHTs, No. (%)		
	FDA (n = 68)	ORCHA (n = 80)	Total (N = 148)
Registered/updated year			
2019	14 (20.6)	10 (12.5)	24 (16.2)
2020	17 (25.0)	9 (11.2)	26 (17.6)
2021	17 (25.0)	15 (18.8)	32 (21.6)
2022	20 (29.4)	46 (57.5)	66 (44.6)
Country of developer			
United States	27 (39.7)	20 (25.0)	47 (31.8)
United Kingdom	1 (1.5)	37 (46.2)	38 (25.7)
China	24 (35.3)	0	24 (16.2)
Other^b^	16 (23.5)	23 (28.8)	39 (26.3)^a^
Type of use			
Over the counter	41 (60.3)	80 (100)	121 (81.8)
Prescription	21 (30.9)	0	21 (14.2)
Both	6 (8.8)	0	6 (4.1)
Target conditions			
Diabetes	35 (51.5)	22 (27.5)	57 (38.5)
Hypertension	22 (32.4)	3 (3.8)	25 (16.9)
COPD	16 (23.5)	4 (5.0)	20 (13.5)
Osteoarthritis	0	3 (3.8)	3 (2.0)
Osteoporosis	0	2 (2.5)	2 (1.4)
Pain management	0	12 (15.0)	12 (8.1)
Healthy living^c^	2 (2.9)	44 (55.0)	46 (31.1)
Intended for ≥2 conditions^d^	3 (4.4)	2 (2.5)	5 (3.4)
Cost^e^			
Entirely free	0	33 (41.2)	33 (22.3)
In-app purchase	0	36 (45.0)	36 (24.3)
Paid	68 (100)	11 (13.8)	79 (53.4)

Abbreviations: COPD, chronic obstructive pulmonary disease; DHTs, digital health technologies; FDA, US Food and Drug Administration; ORCHA, Organisation for the Review of Care and Health Apps.

^a^ The hypothetical patient was female and aged 79 years, with type 2 diabetes, hypertension, chronic COPD, osteoporosis, and osteoarthritis.

^b^ Other countries included Germany (5 DHTs); Canada, Korea, and Switzerland (4 DHTs each); Sweden (3 DHTs); Australia, Denmark, France, Italy, and Japan (2 DHTs each); Cyprus, Iceland, India, Israel, and New Zealand (1 DHT each); and not reported (4 DHTs).

^c^ Includes prescribable DHTs focused on smoking cessation, healthy nutrition, fitness, weight loss, and help for daily life activities adapted to elderly patients.

^d^ DHTs intended to be used for at least 2 conditions among type 2 diabetes, hypertension, chronic obstructive pulmonary disease, osteoporosis, and osteoarthritis.

^e^ Assuming no prescription insurance coverage.

### Abstracting the Elementary Functions

The identified DHTs offered 140 elementary functions to monitor, treat, and/or manage the patient’s conditions (Table 2). The median (IQR) number of elementary functions provided by a DHT was 2 (1-4), with a range of 1 to 22; 38 DHTs (26%) had only 1 function.

**Table 2.** Functions of the Identified DHTs, Overall and by Subgroups Restricted to DHTs Intended for Specific Diseases^a^

Function	Functions, No. (%)							
	All (n = 140)	Diabetes (n = 73)	Hypertension (n = 22)	COPD (n = 42)	Osteoarthritis (n = 22)	Osteoporosis (n = 18)	Pain management (n = 21)	Healthy living (n = 72)
Recording, tracking, or visualizing health-associated parameters	40 (28.6)	23 (31.5)	12 (54.5)	15 (35.7)	7 (31.8)	7 (38.9)	8 (38.1)	20 (27.8)
Providing information or education	18 (12.9)	14 (19.2)	0	10 (23.8)	10 (45.5)	9 (50.5)	3 (14.3)	14 (19.5)
Maintaining motivation	12 (8.6)	2 (1.7)	1 (4.6)	2 (4.8)	1 (4.5)	0	1 (4.8)	7 (9.7)
Communicating with professionals	15 (10.7)	7 (9.6)	3 (13.6)	4 (9.5)	1 (4.5)	0	2 (9.5)	5 (6.9)
Communicating with coaches, peers, and loved ones	15 (10.7)	7 (9.6)	1 (4.6)	1 (2.4)	0	0	3 (14.3)	8 (11.1)
Providing digital therapeutics with just-in-time interventions	8 (5.7)	7 (9.6)	5 (22.7)	6 (14.3)	0	0	0	0
Providing digital therapeutics without just-in-time interventions	23 (16.4)	8 (11.0)	0	4 (9.5)	3 (13.7)	2 (11.1)	4 (19.0)	10 (13.9)
Other functions	9 (6.4)	5 (6.8)	0	0	0	0	0	8 (11.1)

Abbreviations: COPD, chronic obstructive pulmonary disease; DHT, digital health technology.

^a^ For example, the diabetes column presents the functions of devices and apps that were intended for patients with diabetes but may cover functions for pain management or healthy living.

Functions for recording, tracking, or visualizing health-associated parameters were offered by 111 DHTs (75%) (eTable 1 in Supplement 1). This group of functions included both monitoring of physiological parameters (eg, blood glucose) and tracking symptoms, medications, and nutrition using self-reported data. For example, an app that helps patients with diabetes, hypertension, or COPD collect important health information through a built-in noninvasive blood pressure measurement system and inputs for peripheral devices (eg, weight scales).

Functions for providing information or education were offered by 35 DHTs (24%). This group of functions used electronic learning courses, short videos, podcasts, articles, or blogs. For example, we found an app that provides a series of films on the causes, symptoms, and risks of COPD, as well as practical advice on how to recognize and reduce the risk of exacerbations, delivered in the form of interviews with real clinicians and patients.

Functions for maintaining motivation were offered by 34 DHTs (23%). This group of functions included activities to set goals, track progress, or remind patients of care routine (eg, taking measurements or using medications). For example, there was an app to help patients quit smoking by setting goals; providing motivational feedback presenting progress with relevant information, such as how much money they had saved; and sending personalized reminders on the reasons why the patient wanted to stop smoking.

Functions for communicating with health professionals were offered by 30 DHTs (20%). Health professionals could either be a patient’s own health professional or an outside health professionals (eg, from online platforms). For example, there was an app that allows patients with diabetes to connect with a clinician on an online platform who helps them build an individualized treatment plan.

Functions for communicating with coaches, peers, and loved ones were offered by 22 DHTs (15%). For example, we found an app that assigns patients with type 2 diabetes a personal health coach who supports them through a behavior change journey.

Functions providing digital therapeutics with just-in-time interventions (often involving alerts, prompt notifications, or feedback) were offered by 14 DHTs (10%). For example, we found an app that provides coaching messages based on real-time blood glucose values, eg, “Great job monitoring your blood glucose, John. A blood glucose of 210 is high for fasting. Did you eat or drink anything before checking? Did you have clean hands when you pricked your finger?”

Functions providing digital therapeutics without just-in-time interventions were offered by 54 DHTs (37%). Interventions ranged from pharmacological interventions (eg, insulin delivery systems) to cognitive behavioral therapeutics (eg, for smoking cessation), and AI-based chat (eg, for smoking cessation). For example, we found an app that used an AI chatbot to support patients to quit smoking with personalized and human-like conversations.

Other functions were offered by 23 DHTs (16%). These functions included subscribing the blood tests at home, booking appointment with health care practitioners, setting smart shopping lists, or helping patients organize their health schedules (eg, to-do-list activities or clinic schedules).

Of all DHTs, 96 DHTs (65%) involved hardware (eg, wearable devices) and provided 87 elementary functions. There were 52 DHTs (35%) that were standalone apps and provided 103 functions (eTable 2 in Supplement 1).

Among 140 identified elementary functions, 39 (28%) were not considered important, 101 (72%) were considered important by at least 1 health professional, 28 (20%) by at least 3 health professionals, 14 (10%) by at least 4 health professionals, and 7 (5%) by all 5 health professionals. These 7 functions included 1 function for booking appointment with health care practitioners and 6 functions for recording, tracking, or visualizing blood pressure, blood test results, hospital test results (eg, radiology images, examinations), medication intake, physical activities, and symptoms across all conditions. (eTable 3 in Supplement 1).

### Assessing Which DHTs to Prescribe the Hypothetical Patient

We generated 2 prescriptions of DHTs for the hypothetical patient. In the maximalist prescription, the hypothetical patient would need to use 49 DHTs (11 [22%] from FDA databases) to benefit from all 140 elementary functions (eTable 4 in Supplement 1). Only 12 DHTs (25%) in this prescription were entirely free. With this prescription, the hypothetical patient would be required to use at least 43 apps (on smartphone) and 13 devices (some apps can connect to additional devices and some devices can connect to additional apps).

In the parsimonious prescription, the hypothetical patient would need to use 15 DHTs (3 [20%] from FDA databases) to benefit from 28 functions considered important by at least 3 health professionals (Table 3). Only 4 DHTs (27%) in this prescription were entirely free. With this prescription, the hypothetical patient would need to use 13 apps (on smartphone) and 7 to 10 devices (some apps can connect to additional devices) (eFigure 1 in Supplement 1).

**Table 3.** Parsimonious Prescription

DHT (developer)	Target condition	Short description
Zyter RPM (Zyter)^a^	Multiple conditions (type 2 diabetes, hypertension, COPD)	SaMD to RTV blood glucose, BP, heart or pulse rate, oximeter, body temperature, and weight from connected medical devices; transmit data to hospital physician office for review; and send alerts to HCPs when any device reading is out of range. Patients are required to use connected health devices (eg, blood glucose meters, BP cuffs, pulse oximeters, thermometers, digital weight scales, and/or smart watches).
Calcium – Health Guide (Calcium)^a^	Healthy living	App to RTV symptoms, medication intake (all conditions), and health data (medical records) from hospitals and surgery centers, and physical activities and access health improvement programs. Can connect to additional medical devices and fitness devices.
Connected Living by Vodafone (Vodafone Global Enterprise)^a^	Healthy living (for older adults)	App to communicate with their HCPs (about any condition; patients with speech difficulties can use text, images); create appointments and view the shared calendar with their HCPs; access a list of routine activities that are built for them (eg, “Take tablets at 9 am each morning after breakfast”); and use a shopping list to add items that need to be purchased the next time shopping.
Sound Doctor (Digital Trading)	Multiple conditions (type 2 diabetes, hypertension, COPD)	App that provides educational content (courses and film libraries) in long-term conditions (eg, diabetes, COPD, hypertension, back pain, diet and weight management, mental health, stop smoking, sleep, lifestyle).
Diabetes Health Manager (@Point of Care)	Type 2 diabetes	Free app to RTV diabetes symptoms, diabetes medication intake and treatments, and mental health (mood); learn about diabetes care; track progress and receive reminders to maintain motivation; share symptoms with HCPs; and connect to HCPs so they can monitor progress between visits.
Asthma Monitor AM3 G+ (eResearchTechnology)	COPD	Electronic measurement device to RTV lung function (with peak flow meter), COPD symptoms, and COPD medications (self-reported by answering a questionnaire twice a day).
FreeStyle LibreLink – GB (Abbott Diabetes Care)	Type 2 diabetes	Free app to visualize glucose reading, trend arrow, glucose history, and reports (eg, time in range and daily patterns); share data with HCPs and family; and receive low or high glucose alarms. Patients are required to use with FreeStyle Libre or FreeStyle Libre 2 sensors.
Thriva: health checks (Thriva)	Type 2 diabetes	App to order a personalized subscription of finger-prick blood test package at home (eg, liver function, Hb_A1c_ level, vitamin D, cholesterol, thyroid profile, testosterone), visualize blood test results and track progress, receive GP-reviewed results and advice to improve health, and access educational contents (articles, recipes, and podcasts) on how lifestyle habits and blood test results can affect health.
BloodPressureDB (Horst Klier)	Hypertension	App to RTV systolic and diastolic BP, pulse rate, hypertension medications, salt intake, blood glucose, temperature, and weight and share report with HCPs. Patients need to use with connected BP monitoring devices.
AmaraHealth (Priority Digital Health)	Healthy living (smoking cessation, diet and weight loss)	App to track physical activities, mental health (mood), smoking, calories, weight, alcohol intake, water intake, and sleep; learn how to lose weight, quit smoking, care for mental health, and create and maintain a healthy life (eg, articles, videos, audio); set goals and track health data to maintain motivation; access DTx for physical activities (exercise videos) and DTx for mental health care (mindfulness videos); and access healthy recipes.
myfood24 Healthcare (Dietary Assessment)	Healthy living (nutrition)	App to RTV food and drink nutrient intake against targets set by HCPs, maintain motivation on diet/nutrition by tracking progress and receiving reminders to complete food diary, share data with HCPs, and access recipe builder.
Google Fit: Activity Tracker (Google)	Healthy living (fitness)	Free app to RTV all physical activities and maintain motivation on physical activities through setting goals and tracking daily progress (eg, heart points and steps).
MyHealthBoost (Piota Apps)	Healthy living	Group of free apps to access contact information, clinic schedules, and emergency information and communicate with HCPs about any condition by receiving messages from HCPs via targeted push notifications.
Manage My Pain (ManagingLife)	Pain management	App to RTV pain and medications for pain relief (add medication and mark if it helps to manage pain) and share pain reports with HCPs.
Hailie Sensor Nf0107 and Nf0108 (Adherium [NZ])	COPD	Sensor that records and monitors inhaler use (actuation, inspiratory flow, and inhaler shake for prescribed inhaler usage) and sends medication reminders. Patients are required to use with pressurized metered-dose inhalers.

Abbreviations: BP, blood pressure; COPD, chronic obstructive pulmonary disease; DHT, digital health technology; DTx, digital therapeutic; GP, general practitioner; HCP, health care practitioner; RTV, record, track, and/or visualize; SaMD, software as a medical device.

^a^ These devices are all included despite being similar because none offered all functions health professionals considered important.

In the sensitivity analysis, the hypothetical patient would need to use 7 DHTs (1 [14%] from FDA databases) to benefit from 14 functions considered important by at least 4 health professionals (eTable 5 in Supplement 1). Only 2 DHTs (29%) in this prescription were entirely free. With this prescription, the hypothetical patient would need to use 6 apps (on smartphone) and 2 to 6 devices (some apps can connect to additional devices) (eFigure 2 in Supplement 1).

**Figure 2.**
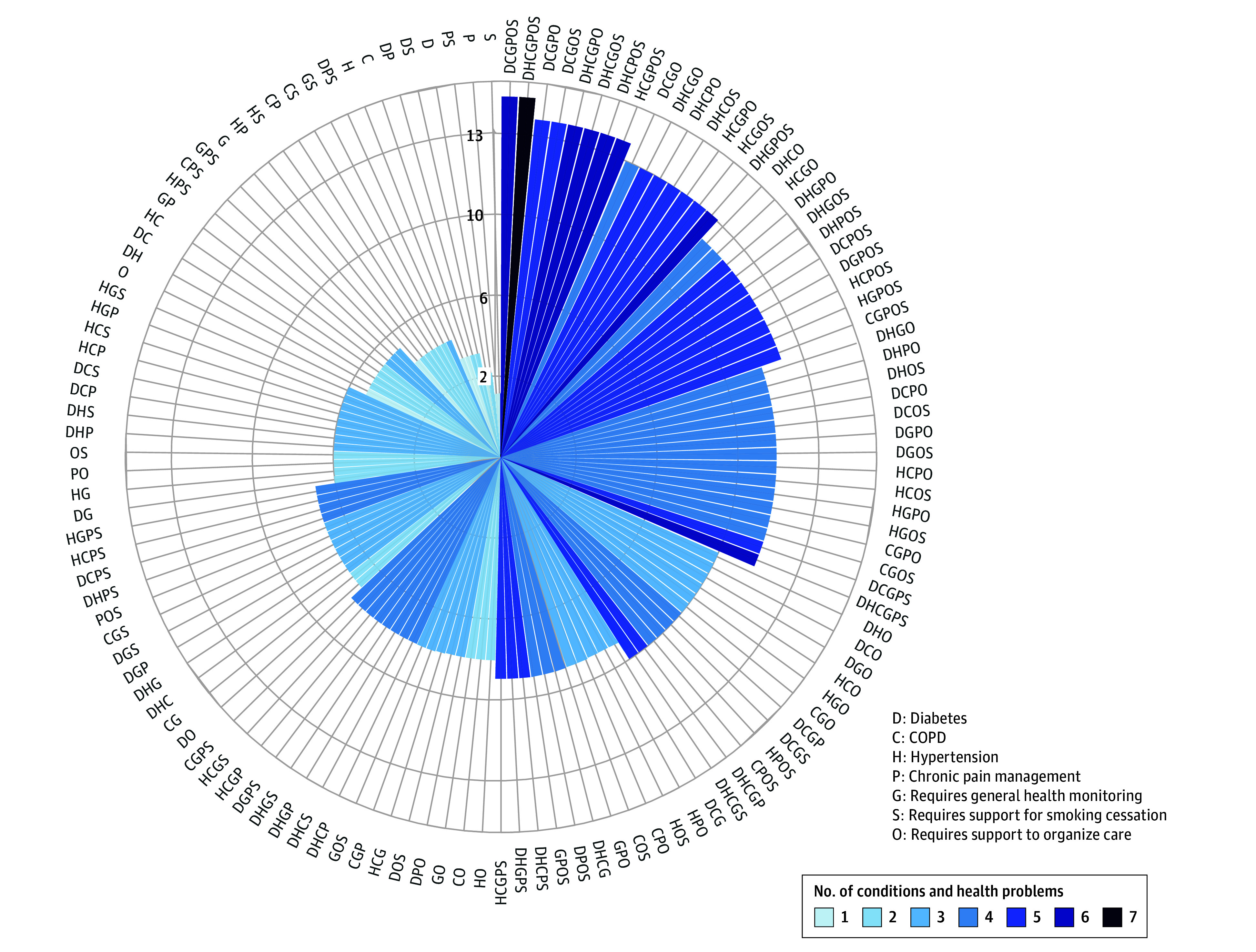
The Accumulation of Digital Health Technologies (DHTs) Used by Hypothetical Patients With Multiple Conditions The figure shows the number of DHTs hypothetical patients who have several conditions (diabetes, hypertension, chronic obstructive pulmonary disease [COPD], and chronic pain management) or health problems (eg, need to stop smoking, need help to organize care) should use to receive benefits from functions at least 3 of 5 health professionals considered important.

Finally, we found that the number of DHTs hypothetical patients would need to use increased quickly when patients had several conditions (diabetes, hypertension, COPD, and chronic pain management) or health problems (eg, need to stop smoking, need help to organize care). For example, a hypothetical patient with hypertension and COPD and who needed help to stop smoking would need to use 6 DHTs to receive benefits from functions at least 3 health professionals considered important (Figure 2).

## Discussion

This systematic review found that a hypothetical patient with 5 chronic conditions would need to use at least 13 apps and 7 devices to benefit from functions health professionals considered important, with 3 apps solely intended for diabetes because none of them covered all functions considered important by health professionals. Our study emphasized the quickly increasing number of apps and devices patients would need to use when their number of conditions or health problems increases, with risks of unintended interactions, alert fatigue, and loss of effectiveness of DHTs. The cumulative burden generated by DHTs is more than their number and potential redundancies: patients may have to familiarize and routinize their lives with different interfaces, connect and manage multiple accounts on online platforms, and potentially deal with contradictory information provided by uncoordinated apps, devices, and people involved (eg, online coaches, clinicians from platforms).

Our research also highlighted the large number and variety of prescribable DHTs. Clinicians willing to use digital medicine would have to choose among 148 DHTs prescribable to the hypothetical patient, including 57 DHTs intended for type 2 diabetes. These figures are only the tip of the iceberg, as our search was focused on the very few DHTs approved by the FDA and/or recommended by ORCHA; that does not compare with the approximately 310 000 health apps available worldwide in 2022.^23^ The number of DHTs identified paralleled the variety of functions they offered; however, not all functions are equal in the eyes of health professionals. Clinicians considered that 28% of functions were not useful and 5% were considered important for most patients with the conditions.

While previous studies have highlighted that DHTs target individual conditions, ours is among the first to demonstrate how this fails to address multimorbidity, with a discrepancy between how DHTs are developed and marketed, ie, within silos and disease-by-disease, and the epidemiological reality of chronic conditions, that is 50% of patients with chronic conditions have multiple chronic conditions.^3,5,12,24,25,26,27^

### Limitations

Our study has several limitations. First, our hypothetical patient lacked contextual details (eg, needs, preferences, digital literacy), which could influence the choice of prescribable DHTs. Second, we considered the hypothetical patient as static, without considering that the prescription of DHTs could be dynamic over time (eg, tracking diabetes parameters only for a few weeks before a consultation). Third, important functions were determined solely by health professionals. We chose this perspective because we focused on prescribable DHTs that could be imposed on patients (as opposed to DHTs chosen by patients). Fourth, our examination relied solely on publicly available information without validation through actual use, although we did consult manufacturers’ websites. This may have led us to miss some functions offered by some DHTs but not emphasized in the product’s description. Fifth, our data came from the US and UK. However, the rapid advancement of digital health (eg, the American College of Physicians has been in collaboration with ORCHA to pilot a centralized app library for the US) should mitigate the impact of geographic differences.

## Conclusions

The findings of this systematic review suggest that the current state of DHTs might generate an important burden for patients with chronic conditions, who often have multiple health problems and conditions. While disease-specific DHTs offer specialized support but may burden patients with multimorbidity, broader DHTs with hundreds of functions are unrealistic and inefficient, as each patient may need only a few functions. The potential solutions include advancing the interoperability of DHTs to create an integrated ecosystem where multiple apps and devices are processed and integrated to harmonize patient interfaces, tasks, alerts, and feedbacks; developing standardized interfaces with recognizable elements to reduce the complexity for patients; promoting communication and education on DHTs for clinicians and patients to enhance their digital health literacy and awareness of DHTs; and following the principles of minimally disruptive digital medicine (eg, clinicians should pay attention to patients’ life circumstances, gradually introduce DHTs by prescribing 1 by 1, assess the usefulness of the digital program regularly, offer timely adjustments).^14,28^ To this end, future DHTs may be designed to include only functions that patients and clinicians considered important and be organized in modules, each covering a condition or function, minimizing the burden to clinicians of responding to data returning from multiple DHTs per patient. Our results also call for comparative information about functions, tasks, and burdens associated with DHTs (eg, need to create an account, need to answer questionnaires) and tools presenting this information to support shared decision-making. It is necessary to change the perspective of digital medicine, often focused on products, to recenter it on patients’ needs and capacities, pursuing minimally disruptive digital medicine.^14^

## Notes

Supplement 1. **eMethods 1.** Search Strategy **eMethods 2.** Exclusion Criteria **eMethods 3.** Selection Process **eMethods 4.** Survey to Assess the Elementary Functions **eTable 1.** Number of Prescribable DHTs by Elementary Function and Condition **eTable 2.** Digital Functions Offered by DHTs Involving Hardware versus DHTs That Are Standalone Apps **eTable 3.** List of Important Functions Health Professionals Considered Important **eTable 4.** Maximalist Prescription **eFigure 1.** DHTs That a Hypothetical Patient With 5 Chronic Conditions Would Need to Use in the Parsimonious Prescription **eTable 5.** Prescription in Sensitivity Analysis **eFigure 2.** DHTs That a Hypothetical Patient With 5 Chronic Conditions Would Need to Use in the Sensitivity Analysis

Supplement 2.
Data Sharing Statement
