# Supplementary material for: Cumulative Burden of Digital Health Technologies for Patients With Multimorbidity: A Systematic Review
Source: JAMA Netw Open. 2025 Apr 25;8(4):e257288. doi: 10.1001/jamanetworkopen.2025.7288 (PMC12032558; doi:10.1001/jamanetworkopen.2025.7288)
Supplement: Supplement 1. — eMethods 1. Search Strategy eMethods 2. Exclusion Criteria eMethods 3. Selection Process eMethods 4. Survey to Assess the Elementary Functions eTable 1. Number of Prescribable DHTs by Elementary Function and Condition eTable 2. Digital Functions Offered by DHTs Involving Hardware versus DHTs That Are Standalone Apps eTable 3. List of Important Functions Health Professionals Considered Important eTable 4. Maximalist Prescription eFigure 1. DHTs That a Hypothetical Patient With 5 Chronic Conditions Would Need to Use in the Parsimonious Prescription eTable 5. Prescription in Sensitivity Analysis eFigure 2. DHTs That a Hypothetical Patient With 5 Chronic Conditions Would Need to Use in the Sensitivity Analysis [file jamanetwopen-e257288-s001.pdf]

# Supplemental Online Content

Phi NTT, Montori VM, Kunneman M, Ravaud P, Tran VT. Cumulative burden of digital health technologies for patients with multimorbidity. *JAMA Netw Open*. 2025;8(4):e257288. doi:10.1001/jamanetworkopen.2025.7288

**eMethods 1.** Search Strategy

**eMethods 2.** Exclusion Criteria

**eMethods 3.** Selection Process

**eMethods 4.** Survey to Assess the Elementary Functions

**eTable 1.** Number of Prescribable DHTs by Elementary Function and Condition

**eTable 2.** Digital Functions Offered by DHTs Involving Hardware versus DHTs That Are Standalone Apps

**eTable 3.** List of Important Functions Health Professionals Considered Important

**eTable 4.** Maximalist Prescription

**eFigure 1.** DHTs that a Hypothetical Patient With 5 Chronic Conditions Would Need to Use in the Parsimonious Prescription

**eTable 5.** Prescription in Sensitivity Analysis

**eFigure 2.** DHTs That a Hypothetical Patient With 5 Chronic Conditions Would Need to Use in the Sensitivity Analysis

This supplemental material has been provided by the authors to give readers additional information about their work.

© 2025 Phi NTT et al. *JAMA Network Open*.

**eMethods 1. Search strategy.**

We systematically searched three FDA databases (i.e., 510(k), Premarket Approval, and De Novo) for approved digital medical devices and searched the ORCHA App library from NHS Somerset for recommended health apps that are prescribable to the hypothetical patient.

***Search strategy in FDA databases***

Three FDA databases included 510(k) Premarket Notification, Premarket Approval, and De Novo of the three regulatory pathways involved in the approval of devices related to the hypothetical patient's diseases:

The 510(k) Premarket Notification Database is located at:
https://www.accessdata.fda.gov/scripts/cdrh/cfdocs/cfpmn/pmn.cfm
The Premarket Approval (PMA) database is located at:
https://www.accessdata.fda.gov/scripts/cdrh/cfdocs/cfPMA/pma.cfm
The De Novo database is located at:
https://www.accessdata.fda.gov/scripts/cdrh/cfdocs/cfPMN/denovo.cfm
When accessing the FDA website of each database, we can find a search interface. We searched the database by the decision date (from 01 January 2019 to 31 December 2022). We chose "Substantial Equivalence (SESE)" for the Decision type in 510(k) database, and "Original Only" for the Supplement Type in PMA database. A given FDA-approved device may be associated with multiple entries. We identified unique devices by checking their names and manufacturers.

In each entry from the search result, different documents are available for download (but are sometimes missing): a product summary redacted by the manufacturer, a decision summary by the FDA and a statement of approval by the FDA. We looked at documents that were available to obtain information for screening.

***Search strategy in ORCHA App library***

ORCHA App Library in the US was being in the pilot stage (in collaboration with American College of Physicians) while there were more completed ORCHA App libraries for each local National Health Service (NHS) in the UK (e.g., NHS Somerset, NHS Lancashire & South Cumbria, or NHS North West London, etc.). Having considered that the difference in the number of health apps among local NHSs was minor, we randomly chose a local NHS App library to review (i.e. ORCHA App library from NHS Somerset). The ORCHA App library from NHS Somerset is located at: https://somerset.orcha.co.uk/.

When accessing the app library, we could find a search interface (by clicking "View filters"). We searched for health apps that were released or updated between 2019 and 2022, under 20 categories that pertain to the health condition of the hypothetical patient:

| Theme | Category |
|---|---|
| Diabetes | Insulin Recording |
| | Type 2 Diabetes |
| Cardiology | High Blood Pressure (Hypertension) |
| Respiratory | COPD |
| Musculoskeletal | Osteoporosis |
| | Osteoarthritis |
| | Arthritis |
| Eye | Diabetic Retinopathy |
| Pain Management | Neck/Shoulder Pain |
| | Hip Pain |
| | Knee Pain |
| | Back Pain |
| | Pain (Generic) |
| | Chronic Pain |
| Healthy Living | Smoking Cessation |
| | Nutrition |
| | Fluid Intake |
| | Fitness |
| | Weight Loss |
| | Caring for Elderly |

© 2025 Phi NTT et al. *JAMA Network Open.*

A given ORCHA-recommended app may be associated with multiple versions. We identified unique apps by checking their operating systems. For each app identified, we downloaded all the available information in the library, including “developer description”, for screening (downloaded on June 12, 2023 by using Data Miner, a free data extraction tool that allows to scrape HTML web pages).

© 2025 Phi NTT et al. *JAMA Network Open*.

**eMethods 2. Exclusion criteria**

We excluded DHTs that were: 1) solely intended for diagnosis; 2) non-connected measurement devices (e.g., non-connected blood glucose monitoring systems); 3) transcutaneous electrical nerve stimulation (TENS) devices; and 4) multi-parameter monitoring devices that monitored less than three out of the following five parameters: blood glucose, blood pressure (systolic and diastolic), heart rate (or pulse rate), pulse oximeter, and weight.

© 2025 Phi NTT et al. *JAMA Network Open*.

**eMethods 3. Selection process**

To select eligible DHTs in the FDA databases, one reviewer (NTTP) screened the 3 FDA databases and excluded entries that did not include any software, such as gloves, gowns, or surgical masks, based on their names. Then, this reviewer downloaded the documents available in the FDA databases (usually the product summary) of the remaining entries and screened the “indications for use” and the “device description” to select the devices that met the eligible criteria. As a given FDA-approved device may be associated with multiple entries (and product summaries), we identified unique devices by checking their names and manufacturers.

To select eligible DHTs in the ORCHA App library, one reviewer (NTTP) screened the “developer description” in 20 categories relevant to the study, and selected the eligible DHTs. As a given ORCHA-recommended DHT may be associated with multiple versions running on different operating systems (i.e., Android, IOS, or website), we identified unique DHTs by checking their operating systems. Whenever the information was unclear about whether the DHT was eligible for the study or not, the reviewer appraised the manufacturer’s website and discussed it with another reviewer (VTT). Finally, the reviewer (NTTP) double checked the names and manufacturers of all included DHTs to ensure each of them was unique.

© 2025 Phi NTT et al. *JAMA Network Open*.

**eMethods 4. Survey to assess the elementary functions.**

To identify functions health professionals considered important, we used a survey, developed by NTTP and an internal medicine specialist (TL, Service de Médecine Interne, AP-HP, Hôpital Européen Georges Pompidou), after several rounds of pilot testing.

The survey included a three-step instruction and with the list of functions without specifying which functions were present in which DHTs. Respondents (health professionals) were first asked to imagine their elderly patients with five conditions similar to the hypothetical patient. Then, they were asked to think of what was essential, in their perspective to prescribe to these patients. Finally, the health professionals were presented with the list of functions included in the identified DHTs and categorized each function into one of the following 3 categories:

1) functions useful for the majority of patients in this situation (important functions);
2) functions useful for specific cases;
3) functions rarely useful for patients in this situation.
Whenever a function was unclear for the health professionals, the researchers would explain it to them.

The five health professionals involved in the survey were: 2 internal medicine specialists (TL and BG, Service de Médecine Interne, AP-HP, Hôpital Européen Georges Pompidou), 1 pulmonologist (AB, Service de Pneumologie, AP-HP, Hôpital Saint-Louis), and 2 general practitioners (SS and AM, Département de Médecine Générale - Faculté de Médecine, Université Paris Cité).

The exact question asked to participants were:

**INSTRUCTIONS**
Step 1.
Imagine that you have elderly patients managing five comorbid diseases:
(1) type 2 diabetes
(2) hypertension
(3) COPD
(4) osteoarthritis
(5) osteoporosis

Step 2.
As a medical doctor, think of what is essential to prescribe to these patients.

Step 3.
Now the landscape of digital medical devices and health apps offers a diverse range of functions tailored to these patients' needs.
In the next sheet, you can see the list of these functions. By marking "X" in the corresponding columns, please choose:
1) functions you believe would be useful to the majority of patients in this situation (important functions),
2) functions you believe would be useful for specific patients (situation-dependent functions),
3) functions you believe would be rarely useful for patients in this situation (optional functions).

(The list of functions enclosed)

© 2025 Phi NTT et al. *JAMA Network Open*.

**eTable 1. Number of prescribable DHTs by elementary function and condition.**

| Functions | All (n = 148) | Diabetes (n = 57) | Hypertension (n = 25) | COPD (n = 20) | Osteoarthritis (n = 3) | Osteoporosis (n = 2) | Pain management (n = 12) | Healthy living (n = 46) |
|---|---|---|---|---|---|---|---|---|
| **Recording/ tracking/ visualizing health associated parameters** | **111 (75.0)** | **46 (80.7)** | **25 (100)** | **17 (85.0)** | **1 (33.3)** | **1 (50.0)** | **7 (58.3)** | **25 (54.3)** |
| Record/ track/ visualize blood glucose | 36 (24.3) | 35 (61.4) | 3 (12.0) | 2 (10.0) | 0 (0.0) | 0 (0.0) | 0 (0.0) | 2 (4.3) |
| Record/ track/ visualize diabetes-related parameters other than blood glucose (i.e., HbA1c, beta-ketone, cholesterol) | 4 (2.7) | 4 (7.0) | 0 (0.0) | 0 (0.0) | 0 (0.0) | 0 (0.0) | 0 (0.0) | 2 (4.3) |
| Record/ track/ visualize insulin data | 9 (6.1) | 9 (15.8) | 0 (0.0) | 0 (0.0) | 0 (0.0) | 0 (0.0) | 0 (0.0) | 1 (2.2) |
| Record/ track/ visualize diabetes symptoms | 3 (2.0) | 3 (5.3) | 1 (4.0) | 1 (5.0) | 0 (0.0) | 0 (0.0) | 0 (0.0) | 0 (0.0) |
| Record/ track/ visualize diabetes medication intake | 2 (1.4) | 2 (3.5) | 0 (0.0) | 0 (0.0) | 0 (0.0) | 0 (0.0) | 0 (0.0) | 0 (0.0) |
| Record/ track/ visualize diabetic retinopathy risk (risk calculation) | 1 (0.7) | 1 (1.8) | 0 (0.0) | 0 (0.0) | 0 (0.0) | 0 (0.0) | 0 (0.0) | 0 (0.0) |
| Record/ track/ visualize blood pressure | 27 (18.2) | 4 (7.0) | 25 (100) | 3 (15.0) | 0 (0.0) | 0 (0.0) | 0 (0.0) | 0 (0.0) |
| Record/ track/ visualize heart rate or pulse rate | 24 (16.2) | 3 (5.3) | 22 (88.0) | 2 (10.0) | 0 (0.0) | 0 (0.0) | 1 (8.3) | 1 (2.2) |
| Record/ track/ visualize salt intake | 1 (0.7) | 0 (0.0) | 1 (4.0) | 0 (0.0) | 0 (0.0) | 0 (0.0) | 0 (0.0) | 0 (0.0) |
| Record/ track/ visualize hypertension medication intake | 1 (0.7) | 0 (0.0) | 1 (4.0) | 0 (0.0) | 0 (0.0) | 0 (0.0) | 0 (0.0) | 0 (0.0) |
| Record/ track/ visualize heart rhythm for risk of cardiac diseases | 3 (2.0) | 0 (0.0) | 3 (12.0) | 0 (0.0) | 0 (0.0) | 0 (0.0) | 0 (0.0) | 0 (0.0) |
| Record/ track/ visualize osteoarthritis symptoms | 1 (0.7) | 0 (0.0) | 0 (0.0) | 0 (0.0) | 1 (33.3) | 1 (50.0) | 0 (0.0) | 0 (0.0) |
| Record/ track/ visualize osteoporosis symptoms | 1 (0.7) | 0 (0.0) | 0 (0.0) | 0 (0.0) | 1 (33.3) | 1 (50.0) | 0 (0.0) | 0 (0.0) |
| Record/ track/ visualize lung function (i.e., spirometry, total lung volume, Forced Expiratory Volume for 1 second, Peak Expiratory Flow, etc.) | 6 (4.1) | 0 (0.0) | 0 (0.0) | 6 (30.0) | 0 (0.0) | 0 (0.0) | 0 (0.0) | 0 (0.0) |
| Record/ track/ visualize respiratory rate | 1 (0.7) | 0 (0.0) | 0 (0.0) | 1 (5.0) | 0 (0.0) | 0 (0.0) | 0 (0.0) | 0 (0.0) |
| Record/ track/ visualize COPD symptoms | 4 (2.7) | 1 (1.8) | 1 (4.0) | 4 (20.0) | 0 (0.0) | 0 (0.0) | 0 (0.0) | 0 (0.0) |
| Record/ track/ visualize abnormal breath sounds (i.e., continuous adventitious breath sounds, wheeze rate) | 1 (0.7) | 0 (0.0) | 0 (0.0) | 1 (5.0) | 0 (0.0) | 0 (0.0) | 0 (0.0) | 0 (0.0) |
| Record/ track/ visualize COPD medication intake | 1 (0.7) | 0 (0.0) | 0 (0.0) | 1 (5.0) | 0 (0.0) | 0 (0.0) | 0 (0.0) | 0 (0.0) |
| Record/ track/ visualize inhaler usage (i.e., actuation, inspiratory flow, inhaler shake, etc.) | 4 (2.7) | 0 (0.0) | 0 (0.0) | 4 (20.0) | 0 (0.0) | 0 (0.0) | 0 (0.0) | 0 (0.0) |
| Record/ track/ visualize symptoms (all conditions) | 2 (1.4) | 0 (0.0) | 0 (0.0) | 0 (0.0) | 0 (0.0) | 0 (0.0) | 1 (8.3) | 1 (2.2) |
| Record/ track/ visualize pain (all pains) | 6 (4.1) | 0 (0.0) | 0 (0.0) | 0 (0.0) | 1 (33.3) | 1 (50.0) | 5 (41.7) | 0 (0.0) |
| Record/ track/ visualize blood test results (all conditions) | 1 (0.7) | 1 (1.8) | 0 (0.0) | 0 (0.0) | 0 (0.0) | 0 (0.0) | 0 (0.0) | 0 (0.0) |

© 2025 Phi NTT et al. *JAMA Network Open.*

| Functions | All (n = 148) | Diabetes (n = 57) | Hypertension (n = 25) | COPD (n = 20) | Osteoarthritis (n = 3) | Osteoporosis (n = 2) | Pain management (n = 12) | Healthy living (n = 46) |
|---|---|---|---|---|---|---|---|---|
| Record/ track/ visualize hospital test results (e.g., radiology images, exams, etc.) | 1 (0.7) | 0 (0.0) | 0 (0.0) | 0 (0.0) | 0 (0.0) | 0 (0.0) | 0 (0.0) | 1 (2.2) |
| Record/ track/ visualize oximetry (i.e., blood oxygen) | 4 (2.7) | 2 (3.5) | 3 (12.0) | 4 (20.0) | 0 (0.0) | 0 (0.0) | 0 (0.0) | 0 (0.0) |
| Record/ track/ visualize body temperature | 3 (2.0) | 2 (3.5) | 3 (12.0) | 2 (10.0) | 0 (0.0) | 0 (0.0) | 0 (0.0) | 0 (0.0) |
| Record/ track/ visualize weight / body mass index | 13 (8.8) | 6 (10.5) | 5 (20.0) | 3 (15.0) | 0 (0.0) | 0 (0.0) | 1 (8.3) | 6 (13.0) |
| Record/ track/ visualize body metrics (e.g., body composition, body circumference, etc.) | 3 (2.0) | 1 (1.8) | 0 (0.0) | 0 (0.0) | 0 (0.0) | 0 (0.0) | 0 (0.0) | 3 (6.5) |
| Record/ track/ visualize diet/ nutrition in general (i.e., log meals, food diary, breakdowns of nutrients, etc.) | 11 (7.4) | 2 (3.5) | 0 (0.0) | 0 (0.0) | 1 (33.3) | 1 (50.0) | 1 (8.3) | 9 (19.6) |
| Record/ track/ visualize carbs intake (for diabetes) | 8 (5.4) | 6 (10.5) | 0 (0.0) | 0 (0.0) | 0 (0.0) | 0 (0.0) | 0 (0.0) | 4 (8.7) |
| Record/ track/ visualize calories (for weight control/ fitness) | 6 (4.1) | 2 (3.5) | 0 (0.0) | 0 (0.0) | 0 (0.0) | 0 (0.0) | 0 (0.0) | 6 (13.0) |
| Record/ track/ visualize water intake | 7 (4.7) | 2 (3.5) | 0 (0.0) | 0 (0.0) | 0 (0.0) | 0 (0.0) | 0 (0.0) | 6 (13.0) |
| Record/ track/ visualize alcohol intake | 2 (1.4) | 1 (1.8) | 0 (0.0) | 0 (0.0) | 0 (0.0) | 0 (0.0) | 0 (0.0) | 2 (4.3) |
| Record/ track/ visualize medication for pain relief (all pains, including musculoskeletal pain) | 3 (2.0) | 0 (0.0) | 0 (0.0) | 0 (0.0) | 0 (0.0) | 0 (0.0) | 3 (25.0) | 0 (0.0) |
| Record/ track/ visualize medication intake (all conditions) | 1 (0.7) | 0 (0.0) | 0 (0.0) | 0 (0.0) | 0 (0.0) | 0 (0.0) | 0 (0.0) | 1 (2.2) |
| Record/ track/ visualize smoking | 2 (1.4) | 0 (0.0) | 0 (0.0) | 0 (0.0) | 0 (0.0) | 0 (0.0) | 0 (0.0) | 2 (4.3) |
| Record/ track/ visualize carbon monoxide in the breath | 2 (1.4) | 0 (0.0) | 0 (0.0) | 2 (10.0) | 0 (0.0) | 0 (0.0) | 0 (0.0) | 2 (4.3) |
| Record/ track/ visualize sleep | 7 (4.7) | 3 (5.3) | 0 (0.0) | 0 (0.0) | 1 (33.3) | 1 (50.0) | 1 (8.3) | 4 (8.7) |
| Record/ track/ visualize mental health (i.e., mindfulness, mood, stress, etc.) | 10 (6.8) | 4 (7.0) | 0 (0.0) | 0 (0.0) | 1 (33.3) | 1 (50.0) | 0 (0.0) | 6 (13.0) |
| Record/ track/ visualize physical activities (i.e., steps, exercises, walking, etc.) | 22 (14.9) | 7 (12.3) | 1 (4.0) | 1 (5.0) | 1 (33.3) | 1 (50.0) | 2 (16.7) | 13 (28.3) |
| Record/ track/ visualize fasting (for weight control/ fitness) | 2 (1.4) | 1 (1.8) | 0 (0.0) | 0 (0.0) | 0 (0.0) | 0 (0.0) | 0 (0.0) | 2 (4.3) |
| **Providing information or education** | **35 (23.6)** | **14 (24.6)** | **0 (0.0)** | **3 (15.0)** | **3 (100)** | **2 (100)** | **7 (58.3)** | **16 (34.8)** |
| Education on diabetes care | 9 (6.1) | 9 (15.8) | 0 (0.0) | 1 (5.0) | 1 (33.3) | 1 (50.0) | 0 (0.0) | 1 (2.2) |
| Education on diabetes care - specific on diet and nutrition | 1 (0.7) | 1 (1.8) | 0 (0.0) | 0 (0.0) | 0 (0.0) | 0 (0.0) | 0 (0.0) | 0 (0.0) |
| Education on diabetes care - specific on physical activity | 1 (0.7) | 1 (1.8) | 0 (0.0) | 0 (0.0) | 0 (0.0) | 0 (0.0) | 0 (0.0) | 0 (0.0) |
| Education on hypertension care | 1 (0.7) | 1 (1.8) | 0 (0.0) | 1 (5.0) | 1 (33.3) | 1 (50.0) | 0 (0.0) | 1 (2.2) |
| Education on COPD care | 2 (1.4) | 1 (1.8) | 0 (0.0) | 2 (10.0) | 1 (33.3) | 1 (50.0) | 0 (0.0) | 1 (2.2) |

© 2025 Phi NTT et al. *JAMA Network Open.*

| Functions | All (n = 148) | Diabetes (n = 57) | Hypertension (n = 25) | COPD (n = 20) | Osteoarthritis (n = 3) | Osteoporosis (n = 2) | Pain management (n = 12) | Healthy living (n = 46) |
|---|---|---|---|---|---|---|---|---|
| Education on smoking cessation | 3 (2.0) | 0 (0.0) | 0 (0.0) | 1 (5.0) | 0 (0.0) | 0 (0.0) | 0 (0.0) | 3 (6.5) |
| Education on weight loss/ healthy weight | 2 (1.4) | 1 (1.8) | 0 (0.0) | 1 (5.0) | 1 (33.3) | 1 (50.0) | 0 (0.0) | 2 (4.3) |
| Education on weight loss/ healthy weight - specific on diet and nutrition | 2 (1.4) | 2 (3.5) | 0 (0.0) | 0 (0.0) | 0 (0.0) | 0 (0.0) | 0 (0.0) | 2 (4.3) |
| Education on weight loss/ healthy weight - specific on body fasting | 2 (1.4) | 0 (0.0) | 0 (0.0) | 0 (0.0) | 0 (0.0) | 0 (0.0) | 0 (0.0) | 2 (4.3) |
| Education on osteoporosis care | 2 (1.4) | 1 (1.8) | 0 (0.0) | 1 (5.0) | 2 (66.7) | 2 (100) | 0 (0.0) | 1 (2.2) |
| Education on osteoarthritis care | 3 (2.0) | 1 (1.8) | 0 (0.0) | 1 (5.0) | 3 (100) | 2 (100) | 0 (0.0) | 1 (2.2) |
| Education on musculoskeletal pain management | 3 (2.0) | 0 (0.0) | 0 (0.0) | 0 (0.0) | 1 (33.3) | 0 (0.0) | 2 (16.7) | 0 (0.0) |
| Education on back pain care | 2 (1.4) | 1 (1.8) | 0 (0.0) | 1 (5.0) | 1 (33.3) | 1 (50.0) | 1 (8.3) | 1 (2.2) |
| Education on pain management (all conditions) | 4 (2.7) | 0 (0.0) | 0 (0.0) | 0 (0.0) | 0 (0.0) | 0 (0.0) | 4 (33.3) | 0 (0.0) |
| Education on diet and nutrition (all conditions) | 8 (5.4) | 4 (7.0) | 0 (0.0) | 1 (5.0) | 1 (33.3) | 1 (50.0) | 0 (0.0) | 8 (17.4) |
| Education on better sleep | 2 (1.4) | 2 (3.5) | 0 (0.0) | 0 (0.0) | 0 (0.0) | 0 (0.0) | 0 (0.0) | 1 (2.2) |
| Education on mental health care | 4 (2.7) | 3 (5.3) | 0 (0.0) | 1 (5.0) | 1 (33.3) | 1 (50.0) | 0 (0.0) | 3 (6.5) |
| Education on general healthy living and lifestyles (all conditions) | 6 (4.1) | 2 (3.5) | 0 (0.0) | 0 (0.0) | 0 (0.0) | 0 (0.0) | 0 (0.0) | 5 (10.9) |
| **Maintaining motivation** (i.e., set goals, track progress, motivational messages, reminders, etc.) | **34 (23.0)** | **9 (15.8)** | **1 (4.0)** | **6 (30.0)** | **1 (33.3)** | **0 (0.0)** | **1 (8.3)** | **21 (45.7)** |
| Motivational support on diabetes care | 6 (4.1) | 6 (10.5) | 0 (0.0) | 0 (0.0) | 0 (0.0) | 0 (0.0) | 0 (0.0) | 0 (0.0) |
| Motivational support on COPD care | 4 (2.7) | 0 (0.0) | 0 (0.0) | 4 (20.0) | 0 (0.0) | 0 (0.0) | 0 (0.0) | 0 (0.0) |
| Motivational support on hypertension care | 1 (0.7) | 0 (0.0) | 1 (4.0) | 0 (0.0) | 0 (0.0) | 0 (0.0) | 0 (0.0) | 0 (0.0) |
| Motivational support on osteoarthritis care | 1 (0.7) | 0 (0.0) | 0 (0.0) | 0 (0.0) | 1 (33.3) | 0 (0.0) | 0 (0.0) | 0 (0.0) |
| Motivational support on pain management program engagement | 1 (0.7) | 0 (0.0) | 0 (0.0) | 0 (0.0) | 0 (0.0) | 0 (0.0) | 1 (8.3) | 0 (0.0) |
| Motivational support on weight control | 3 (2.0) | 0 (0.0) | 0 (0.0) | 0 (0.0) | 0 (0.0) | 0 (0.0) | 0 (0.0) | 3 (6.5) |
| Motivational support on weight loss/ fitness - specific on diet/nutrition | 1 (0.7) | 0 (0.0) | 0 (0.0) | 0 (0.0) | 0 (0.0) | 0 (0.0) | 0 (0.0) | 1 (2.2) |
| Motivational support on diet/ nutrition in general (all conditions) | 3 (2.0) | 0 (0.0) | 0 (0.0) | 0 (0.0) | 0 (0.0) | 0 (0.0) | 0 (0.0) | 3 (6.5) |
| Motivational support on physical activities (all conditions) | 6 (4.1) | 0 (0.0) | 0 (0.0) | 0 (0.0) | 0 (0.0) | 0 (0.0) | 0 (0.0) | 6 (13.0) |
| Motivational support on mental health care | 2 (1.4) | 0 (0.0) | 0 (0.0) | 0 (0.0) | 0 (0.0) | 0 (0.0) | 0 (0.0) | 2 (4.3) |
| Motivational support on smoking cessation | 3 (2.0) | 0 (0.0) | 0 (0.0) | 2 (10.0) | 0 (0.0) | 0 (0.0) | 0 (0.0) | 3 (6.5) |
| Motivational support on practicing positive health behaviors (all conditions) | 5 (3.4) | 3 (5.3) | 0 (0.0) | 0 (0.0) | 0 (0.0) | 0 (0.0) | 0 (0.0) | 5 (10.9) |

© 2025 Phi NTT et al. *JAMA Network Open*.

| Functions | All (n = 148) | Diabetes (n = 57) | Hypertension (n = 25) | COPD (n = 20) | Osteoarthritis (n = 3) | Osteoporosis (n = 2) | Pain management (n = 12) | Healthy living (n = 46) |
|---|---|---|---|---|---|---|---|---|
| **Communicating with professionals** | **30 (20.3)** | **11 (19.3)** | **5 (20.0)** | **5 (25.0)** | **1 (33.3)** | **0 (0.0)** | **4 (33.3)** | **8 (17.4)** |
| Communicate with HCPs about diabetes (i.e., share data/ reports, etc.) | 7 (4.7) | 7 (12.3) | 2 (8.0) | 2 (10.0) | 0 (0.0) | 0 (0.0) | 0 (0.0) | 0 (0.0) |
| Communicate with HCPs about hypertension (i.e., share data, chat, etc.) | 5 (3.4) | 2 (3.5) | 5 (20.0) | 2 (10.0) | 0 (0.0) | 0 (0.0) | 0 (0.0) | 0 (0.0) |
| Communicate with HCPs about COPD (i.e., share data/ reports, video call, etc.) | 2 (1.4) | 1 (1.8) | 1 (4.0) | 2 (10.0) | 0 (0.0) | 0 (0.0) | 0 (0.0) | 0 (0.0) |
| Communicate with HCPs about inhaler usage technique information (i.e., share data/ reports) | 2 (1.4) | 0 (0.0) | 0 (0.0) | 2 (10.0) | 0 (0.0) | 0 (0.0) | 0 (0.0) | 0 (0.0) |
| Communicate with HCPs about pain (any type, including musculoskeletal pain) (i.e., share data/ reports) | 3 (2.0) | 0 (0.0) | 0 (0.0) | 0 (0.0) | 0 (0.0) | 0 (0.0) | 3 (25.0) | 0 (0.0) |
| Communicate with HCPs about diet/ nutrition for all conditions (i.e., share data/ reports) | 1 (0.7) | 0 (0.0) | 0 (0.0) | 0 (0.0) | 0 (0.0) | 0 (0.0) | 0 (0.0) | 1 (2.2) |
| Communicate with HCPs (all conditions) (i.e., sharing data, chat, etc.) | 4 (2.7) | 0 (0.0) | 0 (0.0) | 0 (0.0) | 0 (0.0) | 0 (0.0) | 1 (8.3) | 3 (6.5) |
| Communicate with platform professionals about diabetes (i.e., online consultation, live events, talk, Q&A) | 2 (1.4) | 2 (3.5) | 0 (0.0) | 0 (0.0) | 0 (0.0) | 0 (0.0) | 0 (0.0) | 0 (0.0) |
| Communicate with platform professionals about osteoarthritis personalized treatment | 1 (0.7) | 0 (0.0) | 0 (0.0) | 0 (0.0) | 1 (33.3) | 0 (0.0) | 0 (0.0) | 0 (0.0) |
| Communicate with platform professionals about weight loss (i.e., online consultation, talk, Q&A, etc.) | 1 (0.7) | 1 (1.8) | 0 (0.0) | 0 (0.0) | 0 (0.0) | 0 (0.0) | 0 (0.0) | 0 (0.0) |
| Communicate with platform professionals about diet/ nutrition (i.e., video calls, messages, Q&A, etc.) | 3 (2.0) | 0 (0.0) | 0 (0.0) | 0 (0.0) | 0 (0.0) | 0 (0.0) | 0 (0.0) | 3 (6.5) |
| Communicate with platform professionals about physical activities (i.e., feedback, support, etc.) | 1 (0.7) | 1 (1.8) | 0 (0.0) | 0 (0.0) | 0 (0.0) | 0 (0.0) | 0 (0.0) | 0 (0.0) |
| Communicate with platform professionals about mental health (i.e., Q&A, etc.) | 1 (0.7) | 0 (0.0) | 0 (0.0) | 0 (0.0) | 0 (0.0) | 0 (0.0) | 0 (0.0) | 1 (2.2) |
| Communicate with platform professionals about healthy living | 1 (0.7) | 0 (0.0) | 0 (0.0) | 0 (0.0) | 0 (0.0) | 0 (0.0) | 0 (0.0) | 1 (2.2) |
| Receive GP-reviewed blood test results with evidence-based advice | 1 (0.7) | 1 (1.8) | 0 (0.0) | 0 (0.0) | 0 (0.0) | 0 (0.0) | 0 (0.0) | 0 (0.0) |
| **Communicating with coaches, peers, and loved ones** | **22 (14.9)** | **8 (14.0)** | **1 (4.0)** | **1 (5.0)** | **0 (0.0)** | **0 (0.0)** | **3 (25.0)** | **13 (28.3)** |
| Online health coaching for diabetes | 1 (0.7) | 1 (1.8) | 0 (0.0) | 0 (0.0) | 0 (0.0) | 0 (0.0) | 0 (0.0) | 0 (0.0) |
| Online health coaching for pain management (all pains) | 1 (0.7) | 0 (0.0) | 0 (0.0) | 0 (0.0) | 0 (0.0) | 0 (0.0) | 1 (8.3) | 0 (0.0) |
| Online health coaching for weight loss/ healthy weight - specific on body fasting | 1 (0.7) | 0 (0.0) | 0 (0.0) | 0 (0.0) | 0 (0.0) | 0 (0.0) | 0 (0.0) | 1 (2.2) |

© 2025 Phi NTT et al. *JAMA Network Open*.

| Functions | All (n = 148) | Diabetes (n = 57) | Hypertension (n = 25) | COPD (n = 20) | Osteoarthritis (n = 3) | Osteoporosis (n = 2) | Pain management (n = 12) | Healthy living (n = 46) |
|---|---|---|---|---|---|---|---|---|
| Online health coaching for healthy living (all conditions) | 3 (2.0) | 2 (3.5) | 0 (0.0) | 0 (0.0) | 0 (0.0) | 0 (0.0) | 1 (8.3) | 2 (4.3) |
| Peer support about diabetes care | 2 (1.4) | 2 (3.5) | 0 (0.0) | 0 (0.0) | 0 (0.0) | 0 (0.0) | 0 (0.0) | 0 (0.0) |
| Peer support about healthy diet/ nutrition (all conditions) | 1 (0.7) | 0 (0.0) | 0 (0.0) | 0 (0.0) | 0 (0.0) | 0 (0.0) | 0 (0.0) | 1 (2.2) |
| Peer support about weight loss / fitness (all conditions) | 3 (2.0) | 1 (1.8) | 0 (0.0) | 0 (0.0) | 0 (0.0) | 0 (0.0) | 0 (0.0) | 3 (6.5) |
| Peer support about weight loss/ fitness - specific on diet (e.g., low calorie diet, etc.) | 2 (1.4) | 1 (1.8) | 0 (0.0) | 0 (0.0) | 0 (0.0) | 0 (0.0) | 0 (0.0) | 2 (4.3) |
| Peer support about physical activities (all conditions) | 2 (1.4) | 0 (0.0) | 0 (0.0) | 0 (0.0) | 0 (0.0) | 0 (0.0) | 0 (0.0) | 2 (4.3) |
| Peer support about pain (all conditions) | 1 (0.7) | 0 (0.0) | 0 (0.0) | 0 (0.0) | 0 (0.0) | 0 (0.0) | 1 (8.3) | 0 (0.0) |
| Peer support about smoking cessation | 2 (1.4) | 0 (0.0) | 0 (0.0) | 0 (0.0) | 0 (0.0) | 0 (0.0) | 0 (0.0) | 2 (4.3) |
| Peer support about healthy living (all conditions) | 3 (2.0) | 3 (5.3) | 0 (0.0) | 0 (0.0) | 0 (0.0) | 0 (0.0) | 0 (0.0) | 3 (6.5) |
| Communicate with family about diabetes | 2 (1.4) | 2 (3.5) | 0 (0.0) | 0 (0.0) | 0 (0.0) | 0 (0.0) | 0 (0.0) | 0 (0.0) |
| Communicate with family about hypertension | 1 (0.7) | 0 (0.0) | 1 (4.0) | 0 (0.0) | 0 (0.0) | 0 (0.0) | 0 (0.0) | 0 (0.0) |
| Communicate with family about COPD | 1 (0.7) | 0 (0.0) | 0 (0.0) | 1 (5.0) | 0 (0.0) | 0 (0.0) | 0 (0.0) | 0 (0.0) |
| **Providing digital therapeutics (DTx) with just-in-time interventions** | **14 (9.5)** | **11 (19.3)** | **1 (4.0)** | **4 (20.0)** | **0 (0.0)** | **0 (0.0)** | **0 (0.0)** | **0 (0.0)** |
| Alerts to HCPs out-of-range blood glucose | 2 (1.4) | 2 (3.5) | 1 (4.0) | 1 (5.0) | 0 (0.0) | 0 (0.0) | 0 (0.0) | 0 (0.0) |
| Alerts to HCPs out-of-range blood pressure | 1 (0.7) | 1 (1.8) | 1 (4.0) | 1 (5.0) | 0 (0.0) | 0 (0.0) | 0 (0.0) | 0 (0.0) |
| Alerts to HCPs out-of-range pulse oximeter | 1 (0.7) | 1 (1.8) | 1 (4.0) | 1 (5.0) | 0 (0.0) | 0 (0.0) | 0 (0.0) | 0 (0.0) |
| Alerts to HCPs out-of-range temperature | 1 (0.7) | 1 (1.8) | 1 (4.0) | 1 (5.0) | 0 (0.0) | 0 (0.0) | 0 (0.0) | 0 (0.0) |
| Alerts to HCPs out-of-range weight scale | 1 (0.7) | 1 (1.8) | 1 (4.0) | 1 (5.0) | 0 (0.0) | 0 (0.0) | 0 (0.0) | 0 (0.0) |
| Notification of low/ high blood glucose | 7 (4.7) | 7 (12.3) | 0 (0.0) | 0 (0.0) | 0 (0.0) | 0 (0.0) | 0 (0.0) | 0 (0.0) |
| Feedback on diabetes management based on blood glucose level | 4 (2.7) | 4 (7.0) | 0 (0.0) | 0 (0.0) | 0 (0.0) | 0 (0.0) | 0 (0.0) | 0 (0.0) |
| Feedback to remind patient of important steps of metered dose inhaler use | 3 (2.0) | 0 (0.0) | 0 (0.0) | 3 (15.0) | 0 (0.0) | 0 (0.0) | 0 (0.0) | 0 (0.0) |
| **Providing digital therapeutics (DTx) without just-in-time interventions** | **54 (36.5)** | **17 (29.8)** | **0 (0.0)** | **4 (20.0)** | **2 (66.7)** | **1 (50.0)** | **6 (50.0)** | **28 (60.9)** |
| Personalized insulin dosage recommendation (insulin calculator) | 11 (7.4) | 11 (19.3) | 0 (0.0) | 0 (0.0) | 0 (0.0) | 0 (0.0) | 0 (0.0) | 0 (0.0) |
| Delivery of insulin (i.e., insulin pumps) | 4 (2.7) | 4 (7.0) | 0 (0.0) | 0 (0.0) | 0 (0.0) | 0 (0.0) | 0 (0.0) | 0 (0.0) |
| Decision support tool to manage COPD symptoms | 1 (0.7) | 0 (0.0) | 0 (0.0) | 1 (5.0) | 0 (0.0) | 0 (0.0) | 0 (0.0) | 0 (0.0) |
| High-frequency chest wall oscillation for COPD | 1 (0.7) | 0 (0.0) | 0 (0.0) | 1 (5.0) | 0 (0.0) | 0 (0.0) | 0 (0.0) | 0 (0.0) |

© 2025 Phi NTT et al. *JAMA Network Open*.

| Functions | All (n = 148) | Diabetes (n = 57) | Hypertension (n = 25) | COPD (n = 20) | Osteoarthritis (n = 3) | Osteoporosis (n = 2) | Pain management (n = 12) | Healthy living (n = 46) |
|---|---|---|---|---|---|---|---|---|
| Cognitive behavioral techniques to deal with craving triggers (tobacco, alcohol, and opioids) | 1 (0.7) | 0 (0.0) | 0 (0.0) | 0 (0.0) | 0 (0.0) | 0 (0.0) | 0 (0.0) | 1 (2.2) |
| AI chat on smoking cessation | 1 (0.7) | 0 (0.0) | 0 (0.0) | 0 (0.0) | 0 (0.0) | 0 (0.0) | 0 (0.0) | 1 (2.2) |
| AI chat on nutrition and weight loss | 1 (0.7) | 0 (0.0) | 0 (0.0) | 0 (0.0) | 0 (0.0) | 0 (0.0) | 0 (0.0) | 1 (2.2) |
| Feedback on healthy diet/ nutrition in general (all conditions) | 4 (2.7) | 0 (0.0) | 0 (0.0) | 0 (0.0) | 0 (0.0) | 0 (0.0) | 0 (0.0) | 4 (8.7) |
| Behavior change techniques for self-care using physical activities (multiple conditions) | 1 (0.7) | 1 (1.8) | 0 (0.0) | 0 (0.0) | 0 (0.0) | 0 (0.0) | 0 (0.0) | 0 (0.0) |
| Personalized feedback on patient health and wellness (multiple conditions) | 1 (0.7) | 1 (1.8) | 0 (0.0) | 0 (0.0) | 0 (0.0) | 0 (0.0) | 0 (0.0) | 1 (2.2) |
| Guided physical activity sessions for diabetes care | 1 (0.7) | 1 (1.8) | 0 (0.0) | 0 (0.0) | 0 (0.0) | 0 (0.0) | 0 (0.0) | 0 (0.0) |
| Guide on performing spirometry test (COPD) | 1 (0.7) | 0 (0.0) | 0 (0.0) | 1 (5.0) | 0 (0.0) | 0 (0.0) | 0 (0.0) | 0 (0.0) |
| Guide on respiratory muscle training (COPD) | 1 (0.7) | 0 (0.0) | 0 (0.0) | 1 (5.0) | 0 (0.0) | 0 (0.0) | 0 (0.0) | 0 (0.0) |
| Guide on smoking cessation (i.e., missions to complete, timely and useful advices, etc.) | 1 (0.7) | 0 (0.0) | 0 (0.0) | 0 (0.0) | 0 (0.0) | 0 (0.0) | 0 (0.0) | 1 (2.2) |
| Expert-based programs on weight loss | 2 (1.4) | 1 (1.8) | 0 (0.0) | 0 (0.0) | 0 (0.0) | 0 (0.0) | 0 (0.0) | 2 (4.3) |
| DTx for weight loss/ healthy weight - specific on physical activity (i.e., guided workouts, training programs) | 4 (2.7) | 0 (0.0) | 0 (0.0) | 0 (0.0) | 0 (0.0) | 0 (0.0) | 0 (0.0) | 4 (8.7) |
| DTx for osteoporosis care (i.e., home exercise programs, videos, guided audios, and expert advices) | 1 (0.7) | 0 (0.0) | 0 (0.0) | 0 (0.0) | 1 (33.3) | 1 (50.0) | 0 (0.0) | 0 (0.0) |
| DTx for osteoarthritis care (i.e., home exercise programs, videos, guided audios, and expert advices) | 2 (1.4) | 0 (0.0) | 0 (0.0) | 0 (0.0) | 2 (66.7) | 1 (50.0) | 0 (0.0) | 0 (0.0) |
| DTx for pain management (all conditions) (i.e., training programs, exercises, music therapy, etc.) | 4 (2.7) | 0 (0.0) | 0 (0.0) | 0 (0.0) | 0 (0.0) | 0 (0.0) | 4 (33.3) | 0 (0.0) |
| DTx for musculoskeletal pain management (i.e., physical therapy videos) | 2 (1.4) | 0 (0.0) | 0 (0.0) | 0 (0.0) | 1 (33.3) | 0 (0.0) | 1 (8.3) | 0 (0.0) |
| DTx for back pain care (i.e., exercise programs) | 1 (0.7) | 0 (0.0) | 0 (0.0) | 0 (0.0) | 0 (0.0) | 0 (0.0) | 1 (8.3) | 0 (0.0) |
| DTx for physical activities (all conditions) (i.e., training programs, guided workouts, yoga, walking routes, etc.) | 13 (8.8) | 2 (3.5) | 0 (0.0) | 0 (0.0) | 0 (0.0) | 0 (0.0) | 1 (8.3) | 12 (26.1) |
| DTx for mental health care (i.e., music, guided meditation, breathing exercises, wellness programs, etc.) | 10 (6.8) | 4 (7.0) | 0 (0.0) | 0 (0.0) | 0 (0.0) | 0 (0.0) | 0 (0.0) | 9 (19.6) |
| **Other functions** | **23 (15.5)** | **6 (10.5)** | **0 (0.0)** | **0 (0.0)** | **0 (0.0)** | **0 (0.0)** | **0 (0.0)** | **21 (45.7)** |
| Subscription home blood test (simple finger-prick blood test) | 1 (0.7) | 1 (1.8) | 0 (0.0) | 0 (0.0) | 0 (0.0) | 0 (0.0) | 0 (0.0) | 0 (0.0) |

| Functions | All (n = 148) | Diabetes (n = 57) | Hypertension (n = 25) | COPD (n = 20) | Osteoarthritis (n = 3) | Osteoporosis (n = 2) | Pain management (n = 12) | Healthy living (n = 46) |
|---|---|---|---|---|---|---|---|---|
| Book appointment with HCPs and GPs | 1 (0.7) | 0 (0.0) | 0 (0.0) | 0 (0.0) | 0 (0.0) | 0 (0.0) | 0 (0.0) | 1 (2.2) |
| Inform healthcare organization for any chronic disease (e.g., care plans, appointments, tasks) | 1 (0.7) | 0 (0.0) | 0 (0.0) | 0 (0.0) | 0 (0.0) | 0 (0.0) | 0 (0.0) | 1 (2.2) |
| Smart shopping list for groceries, healthy diets, or specific meal plans | 4 (2.7) | 1 (1.8) | 0 (0.0) | 0 (0.0) | 0 (0.0) | 0 (0.0) | 0 (0.0) | 4 (8.7) |
| Access local healthcare service information | 1 (0.7) | 0 (0.0) | 0 (0.0) | 0 (0.0) | 0 (0.0) | 0 (0.0) | 0 (0.0) | 1 (2.2) |
| Tools for weight loss/ healthy weight - specific on diet and nutrition (i.e., recipes, meal planners) | 8 (5.4) | 2 (3.5) | 0 (0.0) | 0 (0.0) | 0 (0.0) | 0 (0.0) | 0 (0.0) | 8 (17.4) |
| Tools for weight loss/ healthy weight - specific on body fasting (i.e., fasting plans, fasting timer, etc.) | 2 (1.4) | 0 (0.0) | 0 (0.0) | 0 (0.0) | 0 (0.0) | 0 (0.0) | 0 (0.0) | 2 (4.3) |
| Tools for diet and nutrition (all conditions) (i.e., recipes, meal planners, food scanner, etc.) | 12 (8.1) | 4 (7.0) | 0 (0.0) | 0 (0.0) | 0 (0.0) | 0 (0.0) | 0 (0.0) | 11 (23.9) |
| Tools for better sleep (i.e., bedtime stories, music, sleep score, etc.) | 3 (2.0) | 1 (1.8) | 0 (0.0) | 0 (0.0) | 0 (0.0) | 0 (0.0) | 0 (0.0) | 3 (6.5) |

Abbreviations: AI, artificial intelligence; COPD, chronic obstructive pulmonary disease; DHT, digital health technology; DTx, digital therapeutic; GP, general practitioner; HCP, healthcare provider; Q&A, questions and answers.

© 2025 Phi NTT et al. *JAMA Network Open*.

**eTable 2. Digital functions offered by DHTs involving hardware versus DHTs that are standalone apps.**

| List of digital functions provided by DHTs involving hardware |
|---|
| 1. Record/ track/ visualize blood glucose [a]<br>2. Record/ track/ visualize diabetes-related parameters other than blood glucose (i.e., HbA1c, beta-ketone, cholesterol) [a]<br>3. Record/ track/ visualize insulin data [a]<br>4. Record/ track/ visualize diabetes symptoms [a]<br>5. Record/ track/ visualize diabetes medication intake [a]<br>6. Record/ track/ visualize blood pressure [a]<br>7. Record/ track/ visualize heart rate or pulse rate [a]<br>8. Record/ track/ visualize salt intake<br>9. Record/ track/ visualize hypertension medication intake<br>10. Record/ track/ visualize heart rhythm for risk of cardiac diseases [a]<br>11. Record/ track/ visualize lung function (i.e., spirometry, total lung volume, Forced Expiratory Volume for 1 second, Peak Expiratory Flow, etc.) [a]<br>12. Record/ track/ visualize respiratory rate [a]<br>13. Record/ track/ visualize COPD symptoms [a]<br>14. Record/ track/ visualize abnormal breath sounds (i.e., continuous adventitious breath sounds, wheeze rate) [a]<br>15. Record/ track/ visualize COPD medication intake [a]<br>16. Record/ track/ visualize inhaler usage (i.e., actuation, inspiratory flow, inhaler shake, etc.) [a]<br>17. Record/ track/ visualize symptoms (all conditions)<br>18. Record/ track/ visualize pain (all pains)<br>19. Record/ track/ visualize hospital test results (e.g., radiology images, exams, etc.)<br>20. Record/ track/ visualize oximetry (i.e., blood oxygen) [a]<br>21. Record/ track/ visualize body temperature [a]<br>22. Record/ track/ visualize weight / body mass index [a]<br>23. Record/ track/ visualize diet/ nutrition in general (i.e., log meals, food diary, breakdowns of nutrients, etc.)<br>24. Record/ track/ visualize carbs intake (for diabetes) [a]<br>25. Record/ track/ visualize calories (for weight control/ fitness)<br>26. Record/ track/ visualize water intake<br>27. Record/ track/ visualize alcohol intake<br>28. Record/ track/ visualize medication intake (all conditions)<br>29. Record/ track/ visualize smoking<br>30. Record/ track/ visualize carbon monoxide in the breath [a]<br>31. Record/ track/ visualize sleep<br>32. Record/ track/ visualize mental health (i.e., mindfulness, mood, stress, etc.) [a]<br>33. Record/ track/ visualize physical activities (i.e., steps, exercises, walking, etc.) [a]<br>34. Record/ track/ visualize fasting (for weight control/ fitness)<br>35. Education on diabetes care<br>36. Education on smoking cessation [a]<br>37. Education on weight loss/ healthy weight<br>38. Education on weight loss/ healthy weight - specific on diet and nutrition<br>39. Education on pain management (all conditions)<br>40. Education on diet and nutrition (all conditions)<br>41. Education on better sleep<br>42. Education on mental health care<br>43. Education on general healthy living, lifestyles (all conditions)<br>44. Motivational support on diabetes care [a]<br>45. Motivational support on COPD care [a]<br>46. Motivational support on weight control<br>47. Motivational support on weight loss/ fitness - specific on diet/nutrition<br>48. Motivational support on physical activities (all conditions)<br>49. Motivational support on smoking cessation [a]<br>50. Motivational support on practicing positive health behaviors (all conditions)<br>51. Communicate with HCPs about diabetes (i.e., share data/ reports, etc.) [a]<br>52. Communicate with HCPs about hypertension (i.e., share data, chat, etc.) [a]<br>53. Communicate with HCPs about COPD (i.e., share data/ reports, video call, etc.) [a] |

© 2025 Phi NTT et al. *JAMA Network Open*.

| |
|---|
| 54. Communicate with HCPs about inhaler usage technique information (i.e., share data/ reports) [a]<br>55. Communicate with HCPs (all conditions) (i.e., sharing data, chat, etc.)<br>56. Online health coaching for healthy living (all conditions)<br>57. Peer support about weight loss / fitness (all conditions)<br>58. Peer support about weight loss/ fitness - specific on diet (e.g., low calorie, …)<br>59. Peer support about pain (all conditions)<br>60. Peer support about healthy living (all conditions)<br>61. Communicate with family about diabetes<br>62. Communicate with family about hypertension<br>63. Communicate with family about COPD [a]<br>64. Alerts to HCPs out-of-range blood glucose [a]<br>65. Alerts to HCPs out-of-range blood pressure [a]<br>66. Alerts to HCPs out-of-range pulse oximeter [a]<br>67. Alerts to HCPs out-of-range temperature [a]<br>68. Alerts to HCPs out-of-range weight scale [a]<br>69. Notification of low/ high glucose [a]<br>70. Feedback on diabetes management based on blood sugar level<br>71. Feedback to remind patient of important steps of metered dose inhaler use [a]<br>72. Personalized insulin dosage recommendation (calculator) [a]<br>73. Delivery of insulin (i.e., insulin pumps) [a]<br>74. High-frequency chest wall oscillation for COPD [a]<br>75. Personalized feedback on patient health and wellness (multiple conditions)<br>76. Guide on performing spirometry test (COPD)<br>77. Guide on respiratory muscle training (COPD)<br>78. DTx for weight loss/ healthy weight - specific on physical activity (i.e., guided workouts, training programs)<br>79. DTx for pain management (all conditions) (i.e., training programs, exercises, music therapy, etc.)<br>80. DTx for physical activities (all conditions) (i.e., training programs, guided workouts, yoga, walking routes, etc.)<br>81. DTx for mental health care (i.e., music, guided meditation, breathing exercises, wellness programs, etc.)<br>82. Book appointment with HCPs and GPs<br>83. Inform healthcare organization for any chronic disease (e.g., care plans, appointments, tasks)<br>84. Smart shopping list for groceries, healthy diet, or specific meal plans<br>85. Tools for weight loss/ healthy weight - specific on diet and nutrition (i.e., recipes, meal planners)<br>86. Tools for diet and nutrition (all conditions) (i.e., recipes, meal planners, food scanner, etc.)<br>87. Tools for better sleep (i.e., bedtime stories, music, sleep score, etc.) |
| **List of digital functions provided by DHTs that are standalone apps** |
| 1. Record/ track/ visualize blood glucose [a]<br>2. Record/ track/ visualize insulin data [a]<br>3. Record/ track/ visualize diabetic retinopathy risk (risk calculation)<br>4. Record/ track/ visualize blood pressure<br>5. Record/ track/ visualize heart rate or pulse rate<br>6. Record/ track/ visualize osteoarthritis symptoms<br>7. Record/ track/ visualize osteoporosis symptoms<br>8. Record/ track/ visualize COPD symptoms<br>9. Record/ track/ visualize symptoms (all conditions)<br>10. Record/ track/ visualize pain (all pains)<br>11. Record/ track/ visualize blood test results (all conditions)<br>12. Record/ track/ visualize weight / body mass index<br>13. Record/ track/ visualize body metrics (body composition, body circumference, etc.)<br>14. Record/ track/ visualize diet/ nutrition in general (i.e., log meals, food diary, breakdowns of nutrients, etc.)<br>15. Record/ track/ visualize carbs intake (for diabetes)<br>16. Record/ track/ visualize calories (for weight control/ fitness)<br>17. Record/ track/ visualize water intake<br>18. Record/ track/ visualize medication for pain relief (all pains, including musculoskeletal pain)<br>19. Record/ track/ visualize smoking<br>20. Record/ track/ visualize sleep<br>21. Record/ track/ visualize mental health (i.e., mindfulness, mood, stress, etc.)<br>22. Record/ track/ visualize physical activities (i.e., steps, exercises, walking, etc.) |

© 2025 Phi NTT et al. *JAMA Network Open*.

23. Record/ track/ visualize fasting (for weight control/ fitness)
24. Education on diabetes care
25. Education on diabetes care - specific on diet and nutrition
26. Education on diabetes care - specific on physical activity
27. Education on hypertension care
28. Education on COPD care
29. Education on smoking cessation
30. Education on weight loss/ healthy weight
31. Education on weight loss/ healthy weight - specific on diet and nutrition
32. Education on weight loss/ healthy weight - specific on body fasting
33. Education on osteoporosis care
34. Education on osteoarthritis care
35. Education on musculoskeletal pain management
36. Education on back pain care
37. Education on pain management (all conditions)
38. Education on diet and nutrition (all conditions)
39. Education on better sleep
40. Education on mental health care
41. Education on general healthy living, lifestyles (all conditions)
42. Motivational support on diabetes care [a]
43. Motivational support on COPD care
44. Motivational support on hypertension care
45. Motivational support on osteoarthritis care
46. Motivational support on pain management program engagement
47. Motivational support on weight control
48. Motivational support on diet/ nutrition in general (all conditions)
49. Motivational support on physical activities (all conditions)
50. Motivational support on mental health care
51. Motivational support on smoking cessation
52. Motivational support on practicing positive health behaviors (all conditions)
53. Communicate with HCPs about diabetes (i.e., share data/ reports, etc.) [a]
54. Communicate with HCPs about hypertension (i.e., share data, chat, etc.)
55. Communicate with HCPs about pain (any type, including musculoskeletal pain) (i.e., share data/ reports)
56. Communicate with HCPs about diet/ nutrition for all conditions (i.e., share data/ reports)
57. Communicate with HCPs (all conditions) (i.e., sharing data, chat, etc.)
58. Communicate with platform professionals about diabetes (i.e., online consultation, live events, talk, Q&A)
59. Communicate with platform professionals about osteoarthritis personalized treatment
60. Communicate with platform professionals about weight loss (i.e., online consultation, talk, Q&A, etc.)
61. Communicate with platform professionals about diet/ nutrition (i.e., video calls, messages, Q&A, etc.)
62. Communicate with platform professionals about physical activities (i.e., feedback, support, etc.)
63. Communicate with platform professionals about mental health (i.e., Q&A, etc.)
64. Communicate with platform professionals about healthy living
65. Receive GP-reviewed blood test results with evidence-based advice
66. Online health coaching for diabetes
67. Online health coaching for pain management (all pains)
68. Online health coaching for weight loss/ healthy weight - specific on body fasting
69. Online health coaching for healthy living (all conditions)
70. Peer support about diabetes care
71. Peer support about healthy diet/ nutrition (all conditions)
72. Peer support about weight loss / fitness (all conditions)
73. Peer support about physical activities (all conditions)
74. Peer support about smoking cessation
75. Peer support about healthy living (all conditions)
76. Alerts to HCPs out-of-range blood glucose [a]
77. Notification of low/ high glucose [a]
78. Feedback on diabetes management based on blood sugar level [a]
79. Personalized insulin dosage recommendation (calculator) [a]
80. Decision support tool to manage COPD symptoms

© 2025 Phi NTT et al. *JAMA Network Open*.

| |
|---|
| 81. Cognitive behavioral techniques to deal with craving triggers (tobacco, alcohol, and opioids)<br>82. AI chat on smoking cessation<br>83. AI chat on nutrition and weight loss<br>84. Feedback on healthy diet/ nutrition in general (all conditions)<br>85. Behavior change techniques for self-care using physical activities (multiple conditions)<br>86. Guided physical activity sessions for diabetes care<br>87. Guide on smoking cessation (i.e., missions to complete, timely and useful advice)<br>88. Expert-based programs on weight loss<br>89. DTx for weight loss/ healthy weight - specific on physical activity (i.e., guided workouts, training programs)<br>90. DTx for osteoporosis care (i.e., home exercise program, video, guided audio, and expert advices)<br>91. DTx for osteoarthritis care (i.e., home exercise program, video, guided audio, and expert advices)<br>92. DTx for pain management (all conditions) (i.e., training program, exercises, music therapy, etc.)<br>93. DTx for musculoskeletal pain management (i.e., physical therapy video)<br>94. DTx for back pain care (i.e., exercise programs)<br>95. DTx for physical activities (all conditions) (i.e., training programs, guided workouts, yoga, walking route, etc.)<br>96. DTx for mental health care (i.e., music, guided meditation, breathing exercises, wellness program, etc.)<br>97. Subscription home blood test (simple finger-prick blood test)<br>98. Smart shopping list for groceries, healthy diet, or specific meal plans<br>99. Access local healthcare service information<br>100. Tools for weight loss/ healthy weight - specific on diet and nutrition (i.e., recipes, meal planners)<br>101. Tools for weight loss/ healthy weight - specific on body fasting (i.e., fasting plans, fasting timer, etc.)<br>102. Tools for diet and nutrition (all conditions) (i.e., recipes, meal planners, food scanner, etc.)<br>103. Tools for better sleep (i.e., bedtime stories, music, sleep score, etc.) |

Abbreviations: AI, artificial intelligence; COPD, chronic obstructive pulmonary disease; DHT, digital health technology; DTx, digital therapeutic; GP, general practitioner; HCP, healthcare provider; Q&A, questions and answers.

[a] functions that are covered by FDA-approved DHTs.

© 2025 Phi NTT et al. *JAMA Network Open*.

**eTable 3. List of important functions health professionals considered important.**

| | |
|---|---|
| Functions considered important by all 5 health professionals | 1. Record/ track/ visualize blood pressure<br>2. Record/ track/ visualize symptoms (all conditions) [b]<br>3. Record/ track/ visualize medication intake (all conditions) [b]<br>4. Record/ track/ visualize blood test results (all conditions) [b]<br>5. Record/ track/ visualize hospital test results (e.g., radiology images, exams) [b]<br>6. Record/ track/ visualize physical activities (i.e., steps, exercises, walking)<br>7. Book appointments with HCPs and GPs [b] |
| Functions considered important by 4 health professionals | 8. Record/ track/ visualize blood glucose<br>9. Record/ track/ visualize COPD symptoms<br>10. Record/ track/ visualize oximetry (i.e., blood oxygen)<br>11. Record/ track/ visualize weight/ BMI<br>12. Record/ track/ visualize mental health (i.e., mindfulness, mood, stress)<br>13. Notification of low/ high blood glucose<br>14. Access local healthcare service information [b] |
| Functions considered important by 3 health professionals | 15. Record/ track/ visualize diabetes medication intake<br>16. Record/ track/ visualize heart rate or pulse rate<br>17. Record/ track/ visualize hypertension medication intake<br>18. Record/ track/ visualize COPD medication intake<br>19. Record/ track/ visualize inhaler usage (i.e., actuation, inspiratory flow, inhaler shake, etc.)<br>20. Record/ track/ visualize smoking<br>21. Record/ track/ visualize pain (all pains)<br>22. Education on hypertension care<br>23. Education on COPD care<br>24. Motivational support on physical activities (all conditions)<br>25. Motivational support on diet/nutrition in general (all conditions)<br>26. Communicate with HCPs (all conditions)<br>27. Alerts to HCPs out–of–range blood pressure<br>28. Inform healthcare organization for any chronic disease (e.g., care plans, appointments, tasks) |
| Functions considered important by at least 1 health professionals | 29. Record/ track/ visualize diabetes–related parameters other than blood glucose (i.e., HbA1c, beta–ketone, cholesterol)<br>30. Record/ track/ visualize diabetes symptoms<br>31. Record/ track/ visualize insulin data<br>32. Record/ track/ visualize diabetic retinopathy risk (risk calculation)<br>33. Record/ track/ visualize heart rhythm for risk of cardiac diseases<br>34. Record/ track/ visualize respiratory rate<br>35. Record/ track/ visualize lung function (i.e., spirometry, total lung volume, FEV1, PEF, etc.)<br>36. Record/ track/ visualize osteoarthritis symptoms<br>37. Record/ track/ visualize osteoporosis symptoms<br>38. Record/ track/ visualize medication for pain relief (all pains, including musculoskeletal pain)<br>39. Record/ track/ visualize body temperature<br>40. Record/ track/ visualize salt intake<br>41. Record/ track/ visualize water intake<br>42. Record/ track/ visualize alcohol intake<br>43. Record/ track/ visualize sleep<br>44. Record/ track/ visualize fasting (for weight control/ fitness)<br>45. Record/ track/ visualize diet/ nutrition in general (i.e., log meals, food diary, breakdowns of nutrients, etc.)<br>46. Education on diabetes care<br>47. Education on diabetes care – specific on physical activity<br>48. Education on diabetes care – specific on diet and nutrition<br>49. Education on osteoporosis care<br>50. Education on osteoarthritis care<br>51. Education on back pain care |

© 2025 Phi NTT et al. *JAMA Network Open*.

| | |
|---|---|
| | 52. Education on pain management (all conditions)<br>53. Education on smoking cessation<br>54. Education on diet and nutrition (all conditions)<br>55. Education on weight loss/ healthy weight<br>56. Education on weight loss/ healthy weight − specific on diet and nutrition<br>57. Education on weight loss/ healthy weight − specific on body fasting<br>58. Education on mental health care<br>59. Education on general healthy llifestyles (all conditions)<br>60. Motivational support on diabetes care<br>61. Motivational support on hypertension care<br>62. Motivational support on COPD care<br>63. Motivational support on smoking cessation<br>64. Motivational support on weight control<br>65. Motivational support on mental health care<br>66. Communicate with HCPs about diabetes<br>67. Communicate with HCPs about hypertension (i.e., share data, chat, etc.)<br>68. Communicate with HCPs about COPD (i.e., share data, video call, etc.)<br>69. Communicate with HCPs about inhaler usage technique information<br>70. Communicate with HCPs about pain (any type, including musculoskeletal pain)<br>71. Communicate with HCPs about diet/ nutrition for all conditions<br>72. Receive GP−reviewed blood test results with evidence−based advice<br>73. Peer support about diabetes care<br>74. Peer support about smoking cessation<br>75. Peer support about pain (all conditions)<br>76. Peer support about healthy diet/ nutrition (all conditions)<br>77. Peer support about weight loss/ fitness (all conditions)<br>78. Peer support about weightloss/ fitness − specific on diet (e.g., low calorie, etc.)<br>79. Peer support about physical activities (all conditions)<br>80. Peer support about healthy living (all conditions)<br>81. Alerts to HCPs out−of−range blood glucose<br>82. Alerts to HCPs out−of−range temperature<br>83. Alerts to HCPs out−of−range pulse oximeter<br>84. Feedback on diabetes management based on blood sugar level<br>85. Personalized insulin dosage recommendation (calculator)<br>86. Personalized feedback on patient health and wellness (multiple conditions)<br>87. Delivery of insulin (i.e., insulin pumps)<br>88. Decision support tool to manage COPD symptoms<br>89. Guide on smoking cessation (i.e., missions to complete, timely and useful advice, etc.)<br>90. Guided physical activity sessions for diabetes care<br>91. Expert-based programs on weight loss<br>92. DTx for osteoporosis care (i.e., home exercise programs)<br>93. DTx for osteoarthritis care (i.e., home exercise programs)<br>94. DTx for back pain care (i.e., exercise programs)<br>95. DTx for musculoskeletal pain management (i.e., physical therapy videos)<br>96. DTx for pain management (all conditions) (i.e., training programs, exercises, music therapy)<br>97. DTx for physical activities (all conditions) (i.e., training programs, guided workouts, yoga, walking routes, etc.)<br>98. DTx for mental health care (i.e., music, guided meditation, breathing exercises, wellness programs, etc.)<br>99. Subscription of home blood test (simple finger−prick blood test)<br>100. Tools for better sleep (i.e., bedtime stories, music, sleep score, etc.)<br>101. Tools for diet and nutrition (all conditions) (i.e., recipes, meal planner, barcode scanner, etc.) |

Abbreviations: BMI, body mass index; COPD, chronic obstructive pulmonary disease; DHT, digital health technology; DTx, digital therapeutic; FEV1, forced expiratory volume in 1 second; GP, general practitioner; HCP, healthcare provider; HP, health professional; PEF, peak expiratory flow.

[b] functions considered important by at least 4 HCPs not covered by FDA-approved DHTs.

© 2025 Phi NTT et al. *JAMA Network Open.*

**eTable 4. Maximalist prescription.**

| Name of DHT | Target condition | Short description |
|---|---|---|
| Gro Health | Type 2 diabetes | A health app that provides education and behavior change support to help users start and sustain positive health behaviors (e.g. healthy diet, physical activities, or meditations). |
| Zyter Rpm | Multiple conditions (type 2 diabetes, hypertension, COPD) | A SaMD that provides an interface (mobile app for patients and web app for HCPs) with physiological patient monitoring systems (blood glucose, blood pressure, heart rate, pulse oximeter, temperature, and weight). Patients are required to use connected health devices, such as blood glucose meters, blood pressure cuffs, pulse oximeters, thermometers, digital weight scales, and/or smart watches. |
| Carb Manager—Keto Diet Tracker | Diabetes, diet and weight loss | A health app that helps users manage low carb diet by tracking food and macronutrients, tracking exercise and weight, learning about low-carb diets, and connecting with peers. |
| X-PERT Diabetes Digital | Type 2 diabetes | A health app that helps patients learn about the prevention and management of type 2 diabetes. |
| My Arthritis | Osteoarthritis, osteoporosis | A health app that helps patients self-manage their conditions and improve their overall health and wellbeing. |
| Quit Now: My Quit Buddy | Smoking cessation | A health app that helps smokers get and stay smoke-free through tracking progress, receiving daily motivational messages, and connecting with peers. |
| Simple: Intermittent Fasting | Weight loss | A health app that helps users practice healthy eating and intermittent fasting. |
| Sound Doctor | Multiple conditions (type 2 diabetes, hypertension, COPD) | A health app that provides educational contents on long-term conditions: diabetes, COPD, hypertension, back pain, diet and weight management, mental health, stop smoking, sleep, lifestyle, etc. |
| MIR Spirobank | COPD | A health app that helps patients perform spirometry and oximetry test in real-time and directly on smartphone. Patients are required to use with MIR Spirobank Smart (for Spirometry test) or MIR Spirobank Oxi (for Spirometry and Oximetry test). |
| Joint Academy | Osteoarthritis | A health app that provides specific exercises and education to reduce joint pain and increase physical function. |
| Thriva: health checks | Type 2 diabetes | A health app that provides service for home blood test. |
| Lifesum: Healthy Eating | Diet and weight loss | A health app that provides personalized nutrition diets that fit users' lifestyle and taste. |
| Calcium – Health Guide | Healthy living | A health app that pulls users' health data from hospitals and health systems as well as fitness and medical devices, and enables users to track health data and participate in health improvement pathways. |
| Connected Living by Vodafone | Care for elderly | A health app that provides support in daily tasks, e.g., both HCPs and patients can share a list of activities that the patients will do, or patients can create appointments with HCPs. |
| Curable | Pain control | A health app that enables patients to chat with a virtual coach to design a personalized program and learn how to control pain (any condition) through audio lessons and exercises. |
| BreatheSuite MDI Device | COPD | An electronic device intended to attach to the top of the canister of the inhaler to monitor inhaler usage and inhaler technique metrics. |
| Bluestar Rx | Type 2 diabetes | A software system that provides coaching messages based on real-time blood glucose values and trends and helps patients and their HCPs manage diabetes together. |
| KiActiv Health | Type 2 diabetes | A health app that helps patients build daily active habits and learn how physical activities affect their life. |
| Roczen | Type 2 diabetes | A health app that allows users to consult online with specialized clinicians about their diabetes and weight loss management. |

© 2025 Phi NTT et al. *JAMA Network Open*.

| | | |
|---|---|---|
| Diabetes Health Manager | Type 2 diabetes | A health app that helps patients manage diabetes symptoms, medications, etc., connect with their HCPs, and learn latest information about diabetes. |
| BloodPressureDB | Hypertension | A health app that helps patients track/ monitor/ store blood pressure values, manage medications, and share data with HCPs. Patients need to use with connected blood pressure monitoring devices. |
| Breather Coach | COPD | A health app that delivers free respiratory muscle training sessions and helps users track progress. |
| myfood24 Healthcare | Healthy living (nutrition) | A health app that helps users track and analyze food and nutrient intake against their goals. |
| MyFitnessPal: Calorie Counter | Healthy living (nutrition, diet and weight loss) | A health app that helps users track nutrition and activities, learn healthy diets, and connect with peers. |
| Holly Health | Healthy living | A health app that offers 24/7 support and personalized recommendations to achieve users' goals in physical and mental health (e.g., weight loss, sleep management, stress management). |
| World Walking | Healthy weight, healthy living | A health app that provides virtual walk to users to help them walk and keep active in a fun and inspiring way. |
| Manage My Pain | Pain management | A health app that helps patients track pain and medications, and share pain reports with HCPs. |
| FreeStyle LibreLink – GB | Type 2 diabetes | A health app intended to use with FreeStyle Libre and FreeStyle Libre 2 sensors to help users monitor real-time blood glucose values and receive alarms when the glucose is low or high. |
| Curatio: Stronger Together | Type 2 diabetes, healthy living | A health app that provides free coaching and personalized support in managing health conditions (e.g., pre-diabetes, physical activity, weight loss, chronic disease management) through a private community. |
| Hypertension Plus | Hypertension | A health app that helps patients and HCPs remotely monitor patients' blood pressure readings. |
| SmartBP Blood Pressure Tracker | Hypertension | A health app that helps patients track blood pressure measurements and share data with HCPs and family members. |
| NHS Wales: COPDhub | COPD | A health app that provides digital COPD plans and medications to follow, a decision support tool to help manage symptoms, and general education in managing COPD. |
| Quit with Bella | Smoking cessation | A health app that offers a personal coach (advanced artificial intelligence chatbot) and a community of peers. |
| NHS Quit Smoking | Smoking cessation | A health app that offers a 28-day program to quit smoking, including tailored clinical advice. |
| Eat Right Now | Healthy living (nutrition, diet and weight loss) | A health app that delivers step-by-step training to help users create healthy eating habits, e.g., identify stress and emotional eating patterns, or reduce craving-related eating. |
| MyHealthBoost | Healthy living | A group of health apps in which users may access: contacts information, clinic schedules and emergency information. HCPs can message patients via targeted push notifications. |
| Fastic: Intermittent Fasting | Diet and weight loss | A health app that helps users practice intermittent fasting and reach target weight. |
| Pathway through Pain | Pain management | A health app that delivers Pain Management Program for chronic musculoskeletal pain, helping users understand pain and access a combination of physical and psychological therapies. |
| SelfBack | Pain management (back pain) | A health app that provides a tailor-made self-management plan (including exercise program, physical activity tracking, and educational content) to help users manage back pain. |
| Vim Pain Mgmt | Pain management | A health app that helps users track pain, connect with peers, learn about pain management, and access other features (mindfulness activities, exercises, diet recommendations, sleep tools, etc.) |
| RetinaRisk | Diabetic retinopathy | A health app that calculates the diabetic retinopathy risk and enables patients to learn about the key risk factors and mitigation actions to lower the risk. |

© 2025 Phi NTT et al. *JAMA Network Open*.

| | | |
|---|---|---|
| Electromed Smartvest Airway Clearance System | COPD | An electrically powered percussor device that delivers high frequency chest wall oscillation to aid in freeing mucus deposits to improve bronchial drainage and airway clearance. |
| T:Slim X2 Insulin Pump with Interoperable Technology (With T:Connect Mobile App) | Type 2 diabetes | An insulin pump intended for the subcutaneous delivery of insulin, with ability to connect with digitally connected devices (e.g., automated insulin dosing software). |
| iCOquit smokerlyzer | COPD, smoking cessation | A hand-held exhaled breath monitor that detects levels of carbon monoxide on the breath, worked with the iCOquit App to provide visual motivation and track progress. |
| Breathe Easy Mobile Respiratory Monitor (MRM) | COPD | A medical device intended for the spot measurement of respiration rate. |
| Microlife Upper Arm Automatic Digital Blood Pressure Monitor | Hypertension | A non-invasive 24-hour ambulatory blood pressure monitor intended to measure blood pressure, pulse rate, and mean arterial pressure. |
| Wheezo Wheezerate Detector | COPD | A device that contains: (1) wheezo sensing device, (2) wheezo app, and (3) secure cloud server, intended to detect and record abnormal breath sounds at the windpipe (trachea), reported as wheeze rate. |
| Safey Peak Flow Meter | COPD | A medical device that measures PEF and FEV1, connected to Safey App to remind users to take medications and to share information with friends and family members. |
| Asthma Monitor Am3 | COPD | An electronic measurement device to monitor lung function (with peak flow meter), COPD symptoms and medications. |

Abbreviations: COPD, chronic obstructive pulmonary disease; DHT, digital health technology; FEV1, forced expiratory volume in 1 second; HCP, healthcare provider; IAP, in-app purchase; NHS, The National Health Service; PEF, peak expiratory flow; SaMD, Software as a Medical Device.

**eFigure 1. DHTs that a hypothetical patient with 5 chronic conditions would need to use in the parsimonious prescription.**

The figure shows all DHTs that a hypothetical patient would need to use to receive benefits from 28 functions at least 3 out of 5 health professionals considered important.

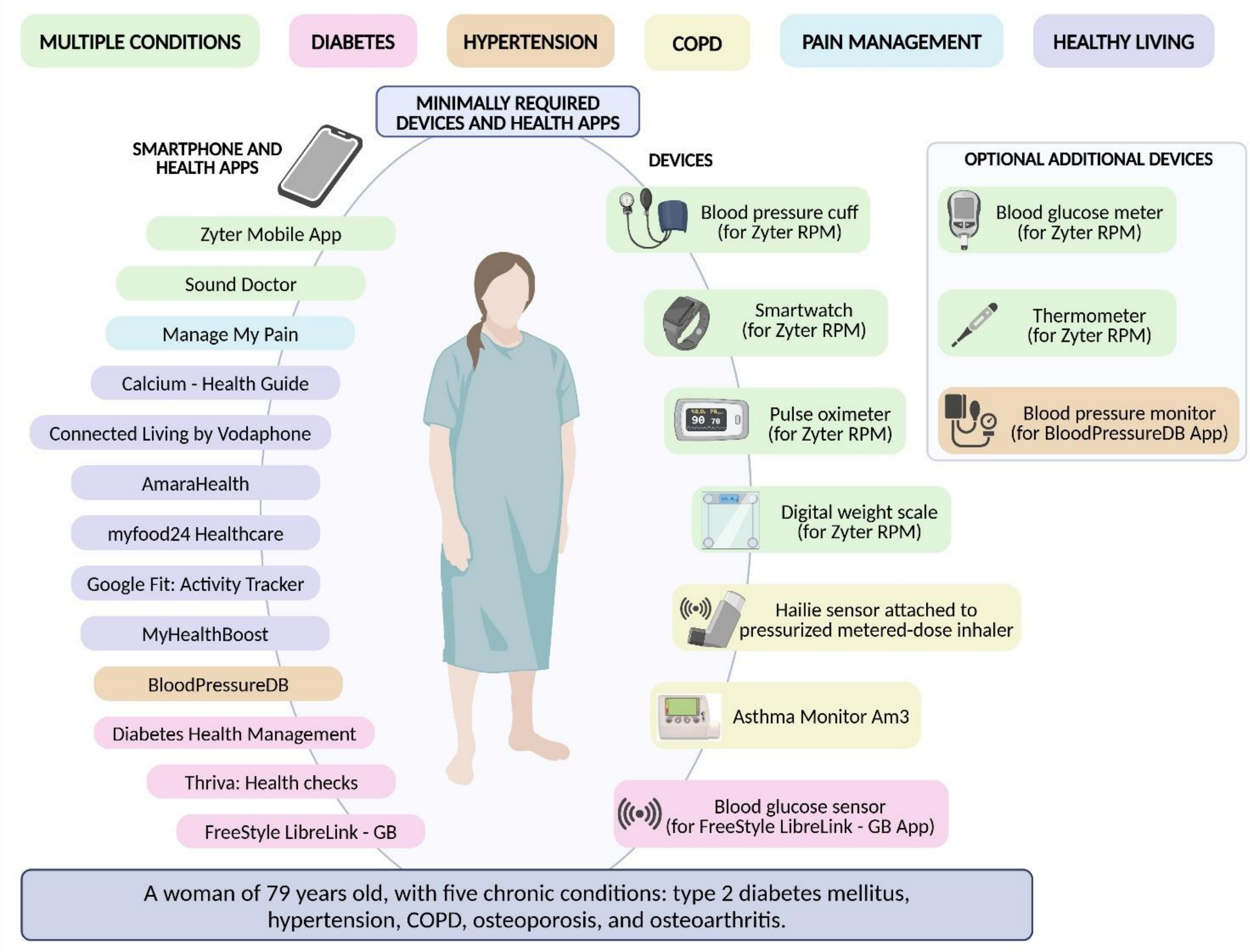


Abbreviations: COPD, chronic obstructive pulmonary disease; DHTs, digital health technologies.

Created in BioRender. Phi, N. (2025) https://BioRender.com/c26m425

**eTable 5. Prescription in sensitivity analysis.**

| Name of DHT | Target condition | Short description |
|---|---|---|
| Commander Flex | Multiple conditions (type 2 diabetes, hypertension, COPD) | An FDA-approved remote monitoring device that enables patients to: 1) record blood pressure, pulse rate, blood glucose, diabetes symptoms, COPD symptoms, pulse oximetry, body temperature, and weight based on a built-in non-invasive blood pressure measurement system and peripheral devices; and 2) send vital sign data to HCPs. Patients are required to use peripheral devices supplied by the manufacturer, such as weight scale, glucose meter, pulse oximeter, and thermometer. |
| Calcium – Health Guide [c] | Healthy living | A health app that enables patients to: 1) record/ track/ visualize symptoms, medication intake (all conditions), health data (medical records) from hospitals and surgery centers, and physical activities; and 2) access health improvement programs. This app can benefit connecting to additional medical devices and fitness devices. |
| Gro health | Type 2 diabetes | A health app that enables patients to: 1) track blood glucose, HbA1c level, heart rate, weight, diet and nutrition (e.g., calories, carbs, fat, and protein), mental health (mood, mindfulness), physical activities, and sleep; 2) access education on healthy food, sleep, wellbeing, and healthy behaviors; 3) set goals and receive notifications to maintain motivation; 4) communicate with a health coach and peer community; 5) receive daily Wellness Score and personalized feedback; 6) access DTx for physical activities (guided exercises), DTx for mental health care (guided meditations); and 7) access recipes, meal plans, and tools for better sleep such as music and bedtime stories. |
| FreeStyle LibreLink – GB | Type 2 diabetes | A free health app that enables patients to: 1) visualize glucose reading, trend arrow, glucose history, and reports (e.g., time in range and daily patterns); 2) share data with HCPs and family; and 3) receive low or high glucose alarms. Patients are required to use with FreeStyle Libre or FreeStyle Libre 2 sensors. |
| Thriva: health checks | Type 2 diabetes | A health app that enables patients to: 1) order a personalized subscription of finger-prick blood test package at home (liver function, HbA1c level, vitamin D, cholesterol, thyroid profile, testosterone, etc.); 2) visualize blood test results and track progress; 3) receive GP-reviewed results and advice to improve health; and 4) access educational contents (articles, recipes, and podcasts) on how lifestyle habits and blood test results can affect health. |
| MyHealthBoost [c] | Healthy living | A group of free health apps that enables patients to: 1) access contact information, clinic schedules, and emergency information; and 2) communicate with HCPs about any condition by receiving messages from HCPs via targeted push notifications. |
| Connected Living by Vodafone [c] | Healthy living (care for elderly) | A health app that enables elderly patients to: 1) communicate with their HCPs (about any condition, patients with speech difficulties can use text, images); 2) create appointments and view the shared calendar with their HCPs; 3) access a list of routine activities that are built for them (e.g., "Take tablets at 9am each morning after breakfast"); and 4) use "shopping list" to add items that need to be purchased the next time shopping. |

Abbreviations: BMI, body mass index; COPD, chronic obstructive pulmonary disease; DHT, digital health technology; DTx, digital therapeutic; FDA, US Food and Drug Administration; GP, general practitioner; HCP, healthcare provider; IAP, in-app purchase.

[c] these devices are all included despite being similar because none offered all functions health professionals considered important.

© 2025 Phi NTT et al. *JAMA Network Open.*

**eFigure 2. DHTs that a hypothetical patient with 5 chronic conditions would need to use in the sensitivity analysis.** The figure shows all DHTs that a hypothetical patient would need to use to receive benefits from 14 functions at least 4 out of 5 health professionals considered important.

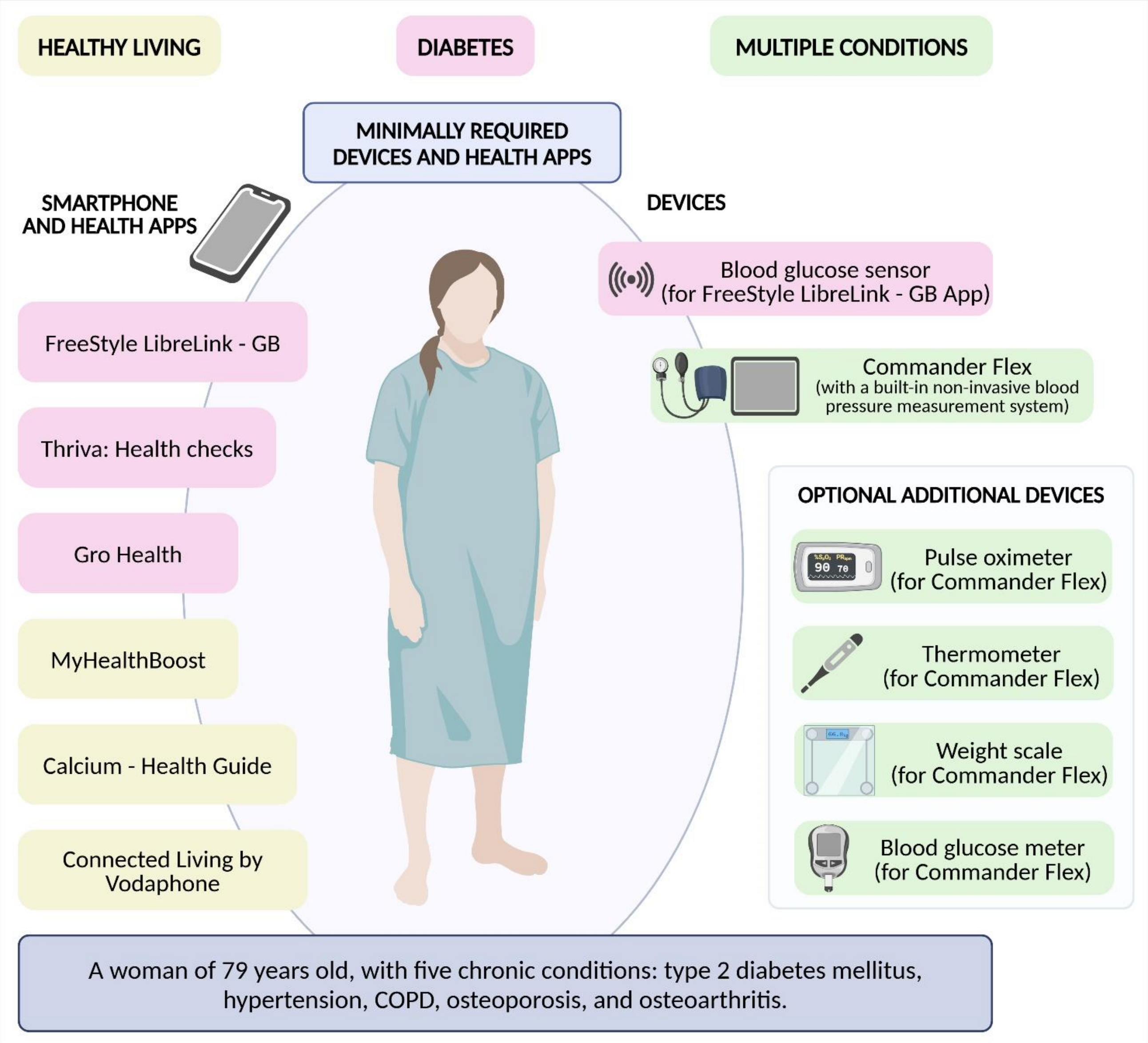


Abbreviations: COPD, chronic obstructive pulmonary disease.

Created in BioRender. Phi, N. (2025) https://BioRender.com/r20u334

© 2025 Phi NTT et al. *JAMA Network Open*.
