# Supplementary material for: Cumulative Burden of Digital Health Technologies for Patients With Multimorbidity: A Systematic Review
Source: JAMA Netw Open. 2025 Apr 25;8(4):e257288. doi: 10.1001/jamanetworkopen.2025.7288 (PMC12032558; doi:10.1001/jamanetworkopen.2025.7288)
Supplement: Supplement 2. — Data Sharing Statement [file jamanetwopen-e257288-s002.pdf]

Data Sharing Statement

Phi. Cumulative Burden of Digital Health Technologies for Patients With Multimorbidity. *JAMA Netw Open*. Published April 25, 2025. doi:10.1001/jamanetworkopen.2025.7288

Data
Data available: Yes
Data types: Data (not involving human participants)
How to access data: The original data from the study are publicly available from the FDA websites and ORCHA App Library from NHS Somerset. Data extracted from FDA product summaries and ORCHA App Library and data extraction grids are available from the corresponding author (email: ngan-thi-thuy.phi@etu.u-paris.fr) upon reasonable request.
When available: With publication

Supporting Documents
Document types: None

Additional Information
Who can access the data: anyone requesting the data
Types of analyses: for any purpose
Mechanisms of data availability: after approval of a proposal
